# Thermodynamic evidence of giant salt deposit formation by serpentinization: an alternative mechanism to solar evaporation

**DOI:** 10.1038/s41598-019-48138-9

**Published:** 2019-08-12

**Authors:** Mathieu Debure, Arnault Lassin, Nicolas C. Marty, Francis Claret, Aurélien Virgone, Sylvain Calassou, Eric C. Gaucher

**Affiliations:** 1 BRGM, French Geological Survey, 45060 Orléans, France; 2 TOTAL, CSTJF, Avenue Larribau, F-64018 Pau Cedex, France

**Keywords:** Geochemistry, Palaeoclimate

## Abstract

The evaporation of seawater in arid climates is currently the main accepted driving mechanism for the formation of ancient and recent salt deposits in shallow basins. However, the deposition of huge amounts of marine salts, including the formation of tens of metres of highly soluble types (tachyhydrite and bischofite) during the Aptian in the South Atlantic and during the Messinian Salinity Crisis, are inconsistent with the wet and warm palaeoclimate conditions reconstructed for these periods. Recently, a debate has been developed that opposes the classic model of evaporite deposition and argues for the generation of salt by serpentinization. The products of the latter process can be called “dehydratites”. The associated geochemical processes involve the consumption of massive amounts of pure water, leading to the production of concentrated brines. Here, we investigate thermodynamic calculations that account for high salinities and the production of soluble salts and MgCl_2_-rich brines through sub-seafloor serpentinization processes. Our results indicate that salt and brine formation occurs during serpentinization and that the brine composition and salt assemblages are dependent on the temperature and CO_2_ partial pressure. Our findings help explain the presence and sustainability of highly soluble salts that appear inconsistent with reconstructed climatic conditions and demonstrate that the presence of highly soluble salts probably has implications for global tectonics and palaeoclimate reconstructions.

## Introduction

Salt deposits are generally thought to form by evaporation in shallow basins with episodic seawater intrusion under dry and hot climate conditions^1^. Salt deposits are considered archives of ancient seawater chemistry, and they are used to track past climate changes^2^, past chemical processes (e.g., the Great Oxidation Event)^3^ or secular variations in seawater chemistry^4,5^. However, evaporation alone cannot explain salt deposits several kilometres thick (salt giants) or deposits of highly soluble evaporites (bischofite, carnallite and tachyhydrite). For example, the dryness required to precipitate the tens of metres of tachyhydrite observed in the South Atlantic is not compatible with the Aptian equatorial climate, which was wet and warm^6,7^, because the calculated equilibrium relative humidity of this salt is as low as 24.4% at 25 °C^8^. Therefore, some authors propose that these thick tachyhydrite layers did not precipitate from normal seawater but most likely originated from hydrothermal interactions within a basaltic host rock^2,9,10^, as supported by the high concentrations of Pb, Zn, Fe, Mn and other trace elements present in the salts^6,11^. Like tachyhydrite, the most soluble potash minerals (carnallite and bischofite) require a dry climate to be preserved, with equilibrium relative humidities of 58.9% for carnallite^12^ and 37.0% for bischofite at 25 °C^13^. Currently, carnallite and bischofite precipitate in the Danakil Depression, but the latter salt quickly deliquesces to sylvite upon exposure to the atmosphere^14^, even in this BWh-type desert climate, according to the Köppen-Geiger climate classification^15^. However, several tens of metres of bischofite are observed in the Sicilian Basin^2^ and in the Gabon and Congo basins^16,17^. The Gabon and Congo salt deposits are dated as Aptian, and thus, bischofite precipitation is not consistent with the wet and warm climate at that time^6,7^. In addition, the Sicilian salt deposits date to the Messinian Salinity Crisis (MSC). Generally, it is suggested that tectonic or climate change occurred during salt deposition^18^. The latter assumption is contradicted by pollen records that reveal no climate change during the MSC and average annual conditions with a temperature of 22 °C (Δ = 15.6–24.7 °C), a relative humidity of 19% (Δ = 17.5–60%) and a rainfall of 575 mm (Δ = 355–872 mm)^19^. Similarly, fossil organic lipids suggest normal salinity (26–34 PSU) and temperatures ranging from 25 to 28 °C during the MSC^20^. Such climatic conditions, if they lasted for years, could be consistent with a decrease in the sea level and thus salt precipitation (if disconnected from the world ocean). However, the conditions were more humid than the current Danakil climate (annual average temperature of 35 °C, 100 mm rain, and relative humidity variations between 23% and 60%) and would have prevented preservation of solid soluble salts (e.g., bischofite) due to seasonal variations and day/night humidity alternations^21–23^.

The observation of highly soluble salts has led to the conclusion that the climate during their deposition was arid on the basis of the evaporation theory^24^. However, in light of the previous results, the amount of soluble salt is not consistent with the palaeoclimate, at least during the Aptian in the Southern Atlantic and during the MSC. In addition, a salt thickness of 1 km requires the evaporation of a 65-km-thick column of seawater^25^. Therefore, reaching the thickness of a salt giant (1–3 km) would require an unrealistic number of filling/evaporation cycles in a shallow basin, without even considering dissolution of previously formed salts by seawater intrusion and the short time of deposition (~500 ka)^10^. Furthermore, the absence of fossils, carbonates or other clastic rocks interbedded within the evaporites is inconsistent with salt formation by evaporation^10,18,26^. A better understanding of the processes of salt deposition may resolve the question of climate dynamics, function and reconstruction. In particular, Quaternary salt deposits are less extensive than ancient salt giants formed during the Aptian or Messinian; is this a result of climate change and greenhouse effects or of other mechanisms?

## Alternative Mechanisms of Salt Production

**Figure 1:**
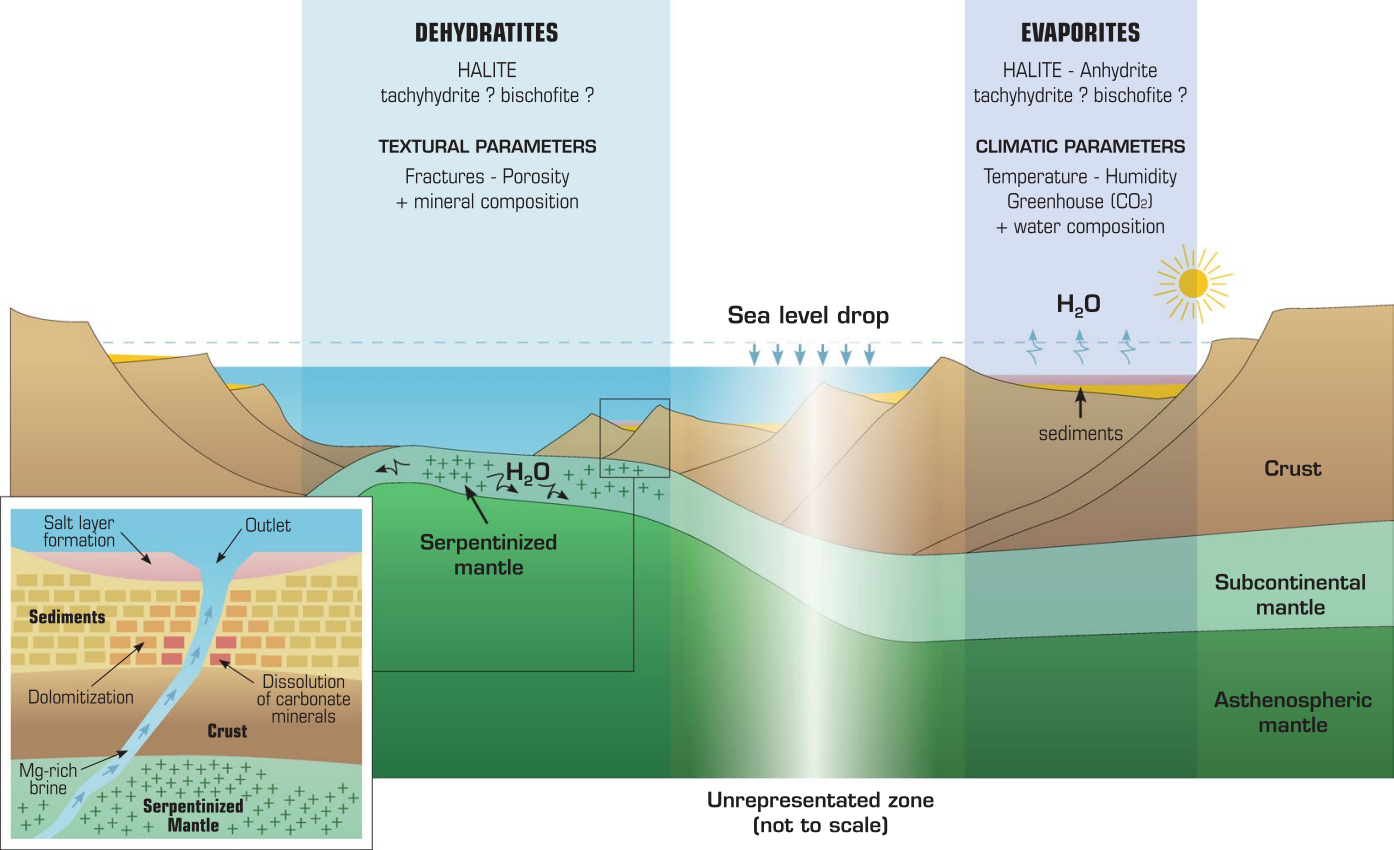
How and where evaporites and dehydratites (salts formed without solar evaporation) form depending on climate and geological contexts. Once formed, Mg-rich brines are able to migrate upward, react with sediments and then precipitate as dolomite or Mg-salts at the bottom of a sea.

Emerging theories propose deep salt formation by supercritical fluids^27^ or exothermic serpentinization in mantle exhumation zones^28–30^. Several thermodynamic processes occur at depth and involve thermal reactions influenced by rifts and/or water consumption by precipitation of hydrated minerals^31^. Seawater infiltrates the crust due to fractures and faults induced by rifting (Fig. 1). When the seawater reaches supercritical conditions (407 °C and 300 bars), water splits off from the NaCl-rich brine. Thus, salts are segregated due to the dependence of solubility on temperature and pressure, with halite on one side and Mg- and Ca-rich salts on the other side, eventually forming the ponds observed today at the bottom of the Red Sea^29^. Serpentinization of mantle rocks produces brines as well. Serpentine contains approximately 13 wt% water; hence, the alteration of 1 m^3^ of peridotite into serpentine by seawater yields 10.5 kg of salt and additional by-products (e.g., magnetite, quartz, and hydrogen^32^). Thus far, theoretical calculations of the amount of salt formed by serpentinization have considered full serpentinization of the minerals constituting the mantle (e.g., olivine and pyroxenes). However, serpentines have a higher molar volume than mantle minerals and cause not only porosity clogging but also fracking, which can propagate serpentinization and thus contribute to enhancing the amount of concentrated brine production. In addition, the fractures caused by the mechanical stress induced by the volume increase^31^ allow the salt-bearing fluids to migrate upward within a hydrothermal plume or by buoyancy^30,33^. The temperature decrease that accompanies the ascent of the brine in the crust and/or the sediments lowers the salt solubility and allows salt deposition.

The mineralogical sequence of salts formed by serpentinization (hereafter referred to as dehydratites) is similar to that observed by seawater evaporation^34^. However, the origin of Mg-rich salt is under debate due to the uptake of Mg in serpentine and the formation of other by-products (e.g., brucite and talc). Nevertheless, it is worth noting that different retrograde processes after serpentinization (as a consequence of oceanic crust ageing) release abundant Mg in the fluid phase, hence favouring the formation of Mg-rich brines or salts, such as bischofite, kainite and carnallite. For example, post-serpentinization processes may produce brucite dissolution and hence the formation of Mg-rich fluids^35^. In addition, post-serpentinization processes produce Mg-rich, high-salinity fluids that can be effective agents in the metasomatism of country rocks. Low-temperature calcite replacing magnesite (and dolomite) previously formed after serpentine also releases aqueous Mg^36^. Some authors disregard the large-scale formation of salt by serpentinization arguing, without thermodynamic calculations, that the fluid resulting from the interaction between seawater and mantle rocks would be depleted in Mg, enriched in K and Ca and finally converted to CaCl_2_-rich brine^37^. To investigate this process, we calculate the processes by using the Pitzer formalism^38^, which is suited to describing salt solubility in complex mixtures of aqueous electrolytes.

## Salt Formation by Serpentinization

**Figure 2:**
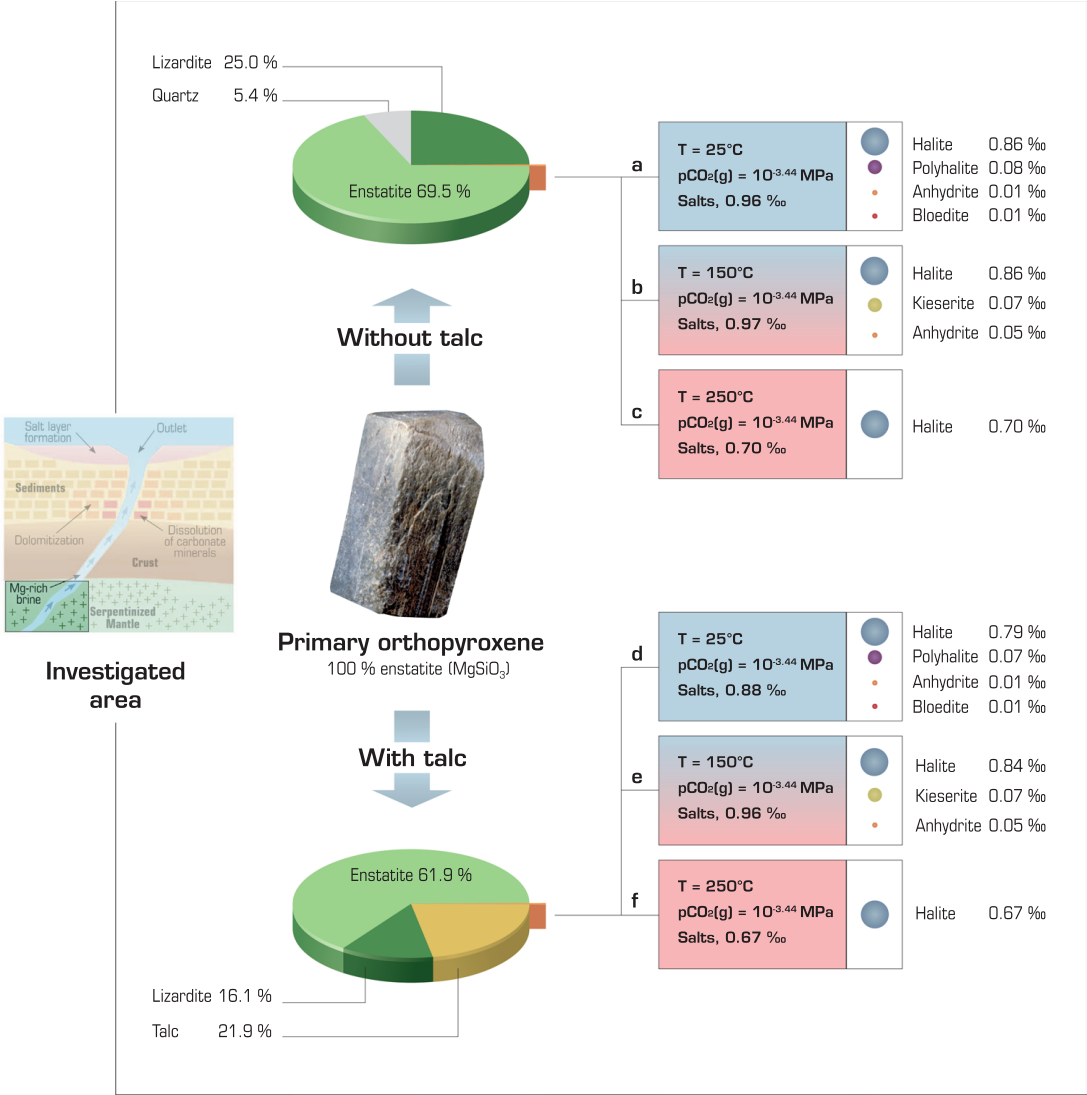
Serpentine (lizardite) and the formation of other secondary phases after Mg-rich orthopyroxene (enstatite) alteration with seawater as a function of temperature at fixed a CO_2_ partial pressure (10^−3^ ^44^ MPa). (**a**) at 25 °C. (**b**) at 150 °C. (**c**) at 250 °C. (**d**) at 25 °C considering talc. (**e**) at 150 °C considering talc. (**f**) at 250 °C considering talc. Salt formation depends on temperature and CO_2_ partial pressure (see Supplementary Information 1). The results are given for the same water consumption (99.0%).

We examine the interaction of Mg-rich orthopyroxene (enstatite) in peridotites with seawater as a function of temperature (25, 150, and 250 °C) and CO_2_ partial pressure (pCO_2_) (free, atmospheric pressure, 1.01325 MPa and 10.1325 MPa) but without considering volumetric expansion consequences on the permeability. We start the calculations with a solid/liquid ratio established according to an initial porosity of 10% (see Methods and Table 1), but serpentinization systematically stops because it completely consumes the water according to the reaction summarized by equation 1. As a result, 70 wt% of pristine orthopyroxene remains unaltered (Fig. 2 and Supplementary Information 1):

1 $$3MgSi{O}_{3}+2{H}_{2}O\to M{g}_{3}S{i}_{2}{O}_{5}{(OH)}_{4}+Si{O}_{2}$$

**Table 1:** Primary and secondary phases considered in the reference calculations. Enstatite is the only primary phase.

Phase	Composition	Temperature dependence	Missing in the Pitzer database	Replaced or added for better temperature dependence*
Enstatite	MgSiO_3_	x		
Quartz	SiO_2_	x		
Lizardite	Mg_3_Si_2_O_5_(OH)_4_		x	x
**#####Salt#####**				
#Chloride				
Halite	NaCl	x		
Sylvite	KCl	x		
Bischofite	MgCl_2_:6H_2_O	x		
MgCl_2_:4H_2_O	MgCl_2_:4H_2_O	x		
MgCl_2_:2H_2_O	MgCl_2_:2H_2_O	x		
Carnallite	KMgCl_3_:6H_2_O	x		
Tachyhydrite	Mg_2_CaCl_6_:12H_2_O		x	x
Antarcticite	CaCl_2_:6H_2_O		x	x
CaCl_2_:4H_2_O	CaCl_2_:4H_2_O		x	x
CaCl_2_:2H_2_O	CaCl_2_:2H_2_O		x	x
CaCl_2_:H_2_O	CaCl_2_:H_2_O		x	x
Kainite	KMgClSO_4_:3H_2_O			x
**#Sulfate**				
Anhydrite	CaSO_4_	x		
Arcanite	K_2_(SO_4_)	x		
Glaserite	Na_2_K_6_(SO_4_)_4_	x		x
Gypsum	CaSO_4_:2H_2_O	x		
Epsomite	MgSO_4_:7H_2_O	x		
Kieserite	MgSO_4_:H_2_O	x		
Mirabilite	Na_2_SO_4_:10H_2_O	x		
Thenardite	Na_2_SO_4_	x		
Polyhalite	K_2_MgCa_2_(SO_4_)_4_:2H_2_O			
Glauberite	Na_2_Ca(SO_4_)_2_	x		x
Hexahydrite	MgSO_4_:6H_2_O	x		
Pentahydrite	MgSO_4_:5H_2_O			
Leonhardite	MgSO_4_:4H_2_O			
**#Carbonates**				
Burkeite	Na_6_CO_3_(SO_4_)_2_			
Calcite	CaCO_3_	x		
Dolomite	CaMg(CO_3_)_2_	x		
Gaylussite	CaNa_2_(CO_3_)_2_:5H_2_O			
Hydromagnesite	Mg_5_(OH)_2_(CO_3_)_4_:4H_2_O	x		
Magnesite	MgCO_3_	x		
Nesquehonite	MgCO_3_:3H_2_O			
Natron	Na_2_CO_3_:10H_2_O			
Na_2_CO_3_	Na_2_CO_3_		x	x
Pirssonite	Na_2_Ca(CO_3_)_2_:2H_2_O	x		x
Thermonatrite	Na_2_CO_3_:H_2_O		x	x
Trona(K)	K_2_NaH(CO_3_)_2_:2H_2_O	x		
Trona	Na_3_H(CO_3_)_2_:2H_2_O			
Bloedite	Na_2_Mg(SO_4_)_2_:4H_2_O	x		
Hanksite	Na_22_K(CO_3_)_2_(SO_4_)9Cl		x	x
**#Hydroxides**				
Brucite	Mg(OH)_2_	x		x
KOH:H_2_O	KOH:H_2_O		x	x
Portlandite	Ca(OH)_2_	x		x
NaOH	NaOH		x	x

^*^In cases where data are missing or incomplete in the Pitzer.dat database, data from the Thermoddem database were considered.

By considering the chemistry of seawater, equation 1 can actually generate a variety of other by-products (as illustrated in Fig. 2). Serpentine is the most important product of the reaction (~25 wt%), followed by quartz (5%) and salts, except under two conditions: at 25 °C and pCO_2_ values of 1.01325 and 10.1325 MPa. CO_2_-rich conditions result in a large quantity of secondary magnesite, with no serpentinization but with dolomite precipitation instead (Fig. S1). When considered, talc precipitates at the expense of quartz (Fig. 2d–f) and is the main by-product.

**Figure 3:**
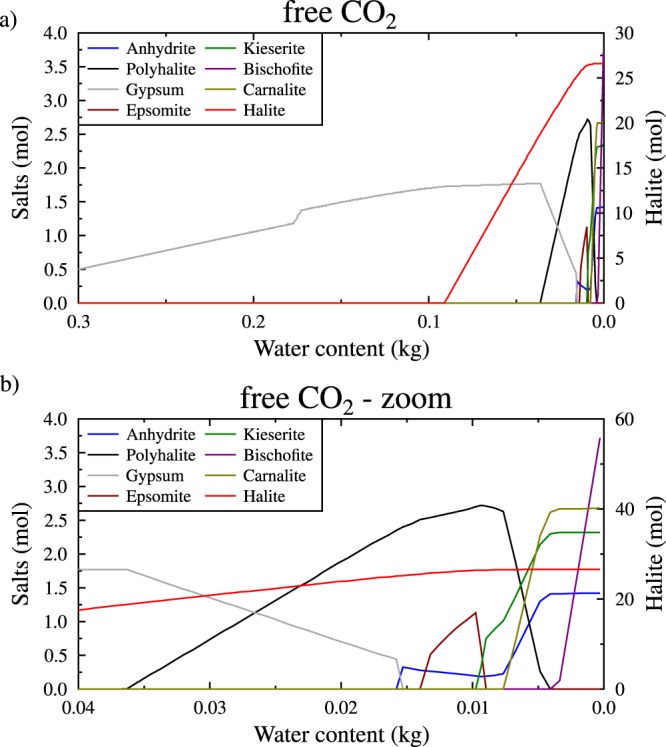
Salts precipitated during serpentinization of Mg-rich orthopyroxene. (**a**) at 25 °C and free pCO_2_ (10^−3.44^ MPa) without talc. (**b**) Magnified view of the highly water-depleted region. The results are the same regardless of whether talc is considered.

The most noticeable by-product is the salt formation occurring in most of the model experiments. The complexity of the final salt assemblage decreases when the temperature and pCO_2_ increase. The salt production appears efficient at 25 and 150 °C, representing 0.1 wt% of the final mineralogical assemblage (Fig. 2a,b,d,e), while at 250 °C, salt represents only 0.07 wt% (Fig. 2c,f). In any case, the major salt is halite. Other salts that precipitate include anhydrite, polyhalite, bloedite, epsomite, kieserite, bischofite, and carnallite. The most complex sequence is observed at 25 °C and free pCO_2_ (10^−3.75^ MPa); under these conditions, salts start to precipitate once 60% of the water is consumed (Fig. 3a and Supplementary Information 2). Some soluble salts can precipitate and re-dissolve as the reaction progresses. For instance, epsomite and polyhalite precipitate before dissolving in favour of bischofite and carnallite (Fig. 3b).

## Brine Formation by Serpentinization

**Figure 4:**
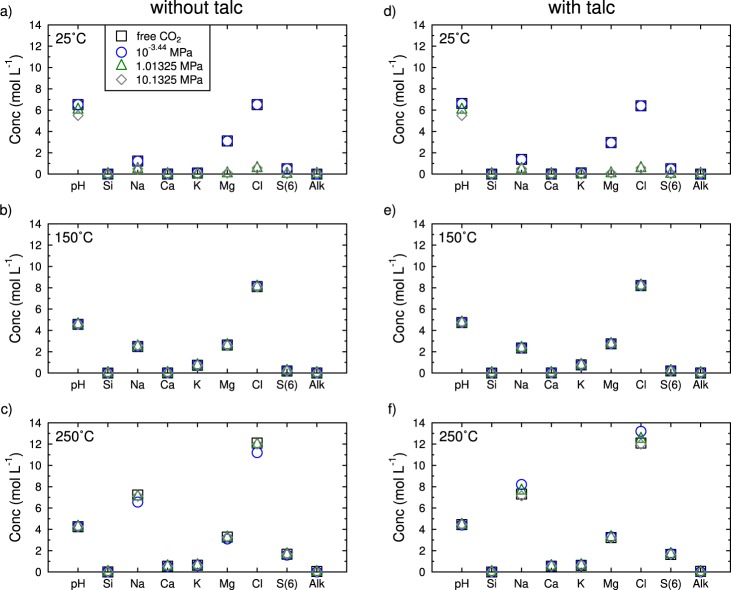
Brine composition once 99.0% of the seawater is consumed by serpentinization. **(a**) at 25 °C without talc and free pCO_2_ equal to 10^−3.23^ MPa. (**b**) at 150 °C without talc and free pCO_2_ equal to 18.4 MPa. (**c**) at 250 °C without talc and free pCO_2_ equal to 114.5 MPa. (**d**) at 25 °C with talc and free pCO_2_ equal to 10^−3.43^ MPa. (**e**) at 150 °C with talc and free pCO_2_ equal to 8.2 MPa. (**f**) at 250 °C with talc and free pCO_2_ equal to 94 MPa. The salinity range varies from 55 to 200 PSU at 25 °C, approximately 210 PSU at 150 °C and from 470 to 530 PSU at 250 °C.

Serpentinization yields highly concentrated fluids, with calculated ionic strengths up to 22 eq·kg^−1^. The brine composition is closely linked to the initial mineralogy, salt solubilities and, particularly, the amount of water remaining after serpentinization has occurred. Indeed, the process consumed 99.9% of the water at 25 °C, 99.5% at 150 °C and 99.0% at 250 °C before calculations numerically stopped. Notwithstanding the higher quantity of water, the ionic strength of the final brine increases with temperature because of the prograde behaviour of most salts (Fig. 4 and Supplementary Information 3). The resulting brines are mostly composed of Cl, Mg and Na (Fig. 4). In some conditions (T = 250 °C), the Ca concentrations reach 0.5 mol kg^−1^ but remain at least 6 times lower than the Na and Mg concentrations (Fig. 4).

In any case, a Na- and Cl-rich brine with a salinity much higher than that of seawater is formed. Although serpentines trap most of the Mg available in the system, the formation of Mg-rich salts is thermodynamically possible, and the Mg concentration in the remaining fluid that stems from serpentinization depends on the CO_2_ partial pressure and temperature applied during the reaction. The most effective conditions for producing MgCl_2_- and CaCl_2_-rich brines are met at 250 °C (Fig. 4c,f).

The nature of the pristine and secondary phases is the other main source of uncertainty and proves to be significant for brine and salt formation. Indeed, considering talc as a by-product leads to another mineralogical assemblage once serpentinization consumes the water. At the end of the reaction, talc, together with serpentine, becomes the main secondary phase. Quartz does not precipitate anymore, but the major salt continues to be halite, with some occurrences of polyhalite, anhydrite, dolomite, magnesite, carnallite, kieserite and bloedite (Fig. 2 and Supplementary Information 1). The thermodynamic calculation does not permit the co-existence of quartz and talc, but salt precipitation still occurs. Both phase assemblages (i.e., with or without talc) are imperfect because talc, like quartz, is actually an extremely minor by-product of serpentinization and is not a major by-product^39,40^. These results highlight the kinetic control of talc precipitation^41^. Considering that talc led to only slight differences in the brine composition, the brine is still enriched in Mg (Fig. 4). In addition, peridotite is not only composed of enstatite since olivine is a major constituent of peridotite as well. Either enstatite or forsterite, shows that the formation of salt is thermodynamically possible when they are in contact with seawater. However, the precipitation of secondary minerals other than salt depends on the phase chosen (calculation not shown), again underlining the need for kinetic control to consider the whole complexity of the peridotite (see calculation methodology in methods).

## Brines at the Bottom of the Seas

**Figure 5:**
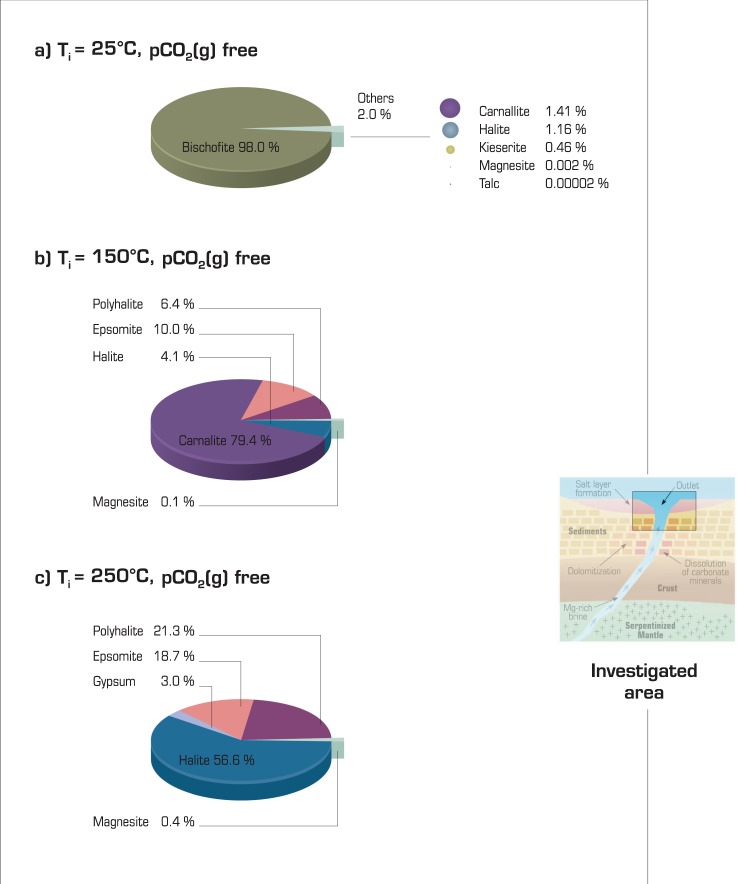
Additional salt precipitation after cooling the brines to a temperature of 4 °C representative of the seafloor. (**a**) Initial temperature of 25 °C and free pCO_2_. (**b**) Initial temperature of 150 °C and free pCO_2_. (**c**) Initial temperature of 250 °C and free pCO_2_. The amount of minerals formed is given in wt%. Tables S1 and S3 in Supplementary Information 3 summarize the initial brine composition.

Once hot brines are formed, they are able to migrate upward through fractures and eventually reach the seafloor^30^. At the seafloor, the temperatures of the brines decrease dramatically due to their contact with seawater. We investigate the chemical consequences of cooling the previously calculated brines down to a temperature representative of the bottom of the ocean (4 °C). In most cases, additional salt precipitated. The exceptions are the two cases that did not display salt formation during the serpentinization event (25 °C and pCO_2_ of 1.01325 and 10.1325 MPa). The composition of the additional salt assemblage is highly dependent on the initial temperature and fluid composition but not on pCO_2_. Thus, bischofite preferentially formed from the brines at 25 °C, carnallite formed from the brines at 150 °C, and halite from the brines at 250 °C (Fig. 5 and Supplementary Information 4). Thus, each final assemblage is representative of the initial temperature of the brines. When considered, talc is insignificant (<0.01% of the additional precipitation) and, unlike magnesite, does not prevent Mg-rich salt precipitation.

The thermodynamic calculations indicate that the temperature decrease due to brine interactions with seawater lowers the salt solubility, which allows the deposition of soluble salt at the seafloor despite the large quantity of water available, as currently observed in pools at the bottom of the Red Sea^28,29^. In addition, cooling of the highly concentrated brine that reaches the seafloor makes it much denser than seawater. Consequently, a layer of solute-rich brine remains above the seafloor and preserves newly formed salts from dissolution. Except for the brine formed at 250 °C, the salt formed by this process on the seafloor contains a lower proportion of halite than of other soluble salts. This result can be considered a discriminating criterion of this type of salt formation process compared to solar evaporation.

In summary, the combination of the serpentinization process and the upward migration of concentrated brine to the seafloor results in the following mass balance estimate. A quantity of seawater that contains 1000 kg of pure water produces approximately 20–27 kg of halite (during serpentinization), 100 g to 4 kg of highly soluble salt and up to 4 kg of additional halite (on the seafloor) depending on the temperature and CO_2_ level. These results are perfectly in line with the observations at the bottom of the Red Sea, where Mg- and Ca-rich salts settle in ponds and halite is left at depth due to salt segregation^29^. Here, serpentine represents 30% of the pristine Mg-rich orthopyroxene at 2.9 m^3^ (or 9.2 tons) considering 1000 kg of pure water. Considering the amount of serpentinized rock estimated in the Central Western Iberian margin (3500 × 10^9^ tons)^31^, up to 1.5 × 10^9^ tons (or 7.0 × 10^8^ m^3^) of soluble salts may have settled on the seafloor. If we consider the average rate of serpentinization (42%) estimated by Pinto *et al*.^31^, the amount of salt produced would be approximately 9.9 × 10^8^ m^3^. The total amount of salt formed is even higher when considering the additional halite precipitation. Therefore, the formation of giant salt deposits by serpentinization and upward migration of brine appears to be achievable.

## Implications for Global Tectonics and Palaeoclimate Reconstruction

The composition of the mineralogical assemblage depends on the mineral solubilities, which are related to the physicochemical conditions. First, the formation of soluble salt (bischofite) and brine (rich in MgCl_2_ or CaCl_2_) is thermodynamically possible during serpentinization at temperatures ranging from 25 to 250 °C. As serpentinization is usually thought to occur at high temperatures and pressures, the investigated temperature range may appear low, but the serpentinization temperature depends on the depth at which the reaction occurs, and the reaction can actually occur at ambient temperature^32,42–44^. The low water diffusion coefficient (10^−7^–10^−8^ cm^2^ s^−1^ at 34 °C) can limit the rate of serpentinization below 100 °C, whereas serpentinization is more efficient at 300 °C (1 km formed in 1 Ma)^45^. However, the temperature of formation of lizardite and chrysotile can range from 85 to 185 °C, while the temperature of formation of antigorite ranges from 220 to 460 °C^44^. Because high-salinity geochemical databases are not reliable beyond 250 °C, our study focuses on lizardite.

Second, salt formation depends on temperature and pCO_2_. Because serpentinization occurs at a large range of temperatures and the amount of CO_2_ released by the mantle is not well constrained, we investigated the possible ranges of the two parameters. The results show that (1) salt and brines form in all cases, but their composition varies with temperature and pCO_2_, (2) low pCO_2_ and low temperature enhance soluble salt precipitation, (3) a high temperature does not necessarily produce more salt (salts are generally more soluble at high temperatures) but does produce more concentrated brines, and (4) additional soluble salt formation occurs when seawater cools the brines close to the seafloor. The quantity of precipitated salt is higher when the initial brines formed at high temperatures.

After salt generation, hydrothermal flow can carry the salt or brine upwards from depth and deposit solid salts onto the seafloor upon cooling in ponds of high-density brines^28,29^. During its travel to the seafloor, the Mg-rich fluid can react with carbonates (Fig. 1) or other Ca-rich minerals and can become enriched in Ca, as observed in hydrothermal vents and hot springs^28,29^. The upwelling of hot CaCl_2_ brines has been reported in studies that did not explain their origins, but hypothesizing interactions of seawater with mantle rocks and basalts^11^. In our view, the serpentinization process can be one of the possible mechanisms explaining this hot brine formation at depth. In this situation, CaCl_2_ is produced either as a by-product of the reaction (e.g., diopside instead of enstatite as the main reactant) or by MgCl_2_ brines that encounter carbonates (which are present in the southern Atlantic sub-seafloor) during upward migration, dissolve/transform them (dolomitization) and become enriched in Ca. In addition, turbidites are possibly associated with salt rock deposits. In all cases, serpentinization increases the water salinity^46^, and the reactions can be traced in hydrothermal vents by Ca and Mg enrichment of the fluid^47^. However, the calculated amount of salt that can form by serpentinization does not consider the volumetric expansion induced by serpentine precipitation. Considering this mechanism leads to two competitive behaviours: on the one hand, pore clogging could slow the serpentinization process, while on the other hand, fractures and faults induced by crystallization pressure phenomena could sustain the serpentinization process^48,49^. Moreover, a comparison with the amount of estimated serpentinized mantle in the Central Western Iberian margin^31^ shows the ability of the reaction to form giant salt deposits. In summary, giant salt deposits likely involve several processes, one of which is evaporation, but the contribution of serpentinization cannot be discarded as long as the amount of salt formed by each mechanism is not quantified; in any case, salt deposition is also influenced by plate tectonics (Wilson cycles)^29^. However, we show that (1) the deposition of salt rocks does not require the closure of the basin and large quantities of surficial saltwater are no longer mandatory and (2) salt rock deposits can appear in the deepest part of a marine basin. Therefore, the salt base is not necessarily flat, as the dehydratite deposits could be syn-rift deposits and could be shaped by the geological structure.

Palaeoclimate reconstruction relies on the nature of sedimentary rocks (e.g., tillites and evaporites), sedimentology (e.g., transgression and regression cycles), fossils, isotopic data (e.g., oxygen and carbon) and Wilson cycles. Thus, considering evaporation to be the sole possible process of the formation of soluble salts may lead to contradictory conclusions regarding the climate at a particular time. In other words, the presence of salt rocks alone is not necessarily an indicator of past climate and is not synonymous with palaeo-aridity. Therefore, the debate between evaporation and serpentinization processes as the genetic mechanism of salt giants and soluble salt precipitation has implications far beyond the depositional processes, as its resolution will help to better constrain the palaeoclimate. Therefore, gaining insight into the processes responsible for soluble salt deposition will enhance our understanding of palaeoclimate and climatic models.

## Methods

Below, we summarize the methodology applied to obtain the results presented in this article.

### Thermodynamic model

The geochemical code PhreeqC and the Pitzer database^50^ were used in the study. All calculations were performed at thermodynamic equilibrium. When available in the Thermoddem database^51^, missing phases or better temperature dependences were added or replaced in the Pitzer database. The seawater composition was taken from Nordstrom *et al*.^52^.

We checked the consistency of the phases with the temperature: bischofite precipitates below 117 °C, MgCl_2_:4H_2_O is considered above 117 °C up to 185 °C, and MgCl_2_:2H_2_O is considered beyond 185 °C^53^. Carnallite is observed below 200 °C^53^. Antarcticite is observed below 31 °C, CaCl_2_:4H_2_O precipitates above 31 °C up to 45 °C, then CaCl_2_:2H_2_O precipitates up to 176 °C, while CaCl_2_:H_2_O is considered beyond 176 °C^53^. Kainite is observed until 70 °C and does not precipitate above this temperature^54^.

### Database limit

The validity of thermodynamic databases used for geochemical modelling in high-salinity conditions does not exceed 250 °C. Therefore, serpentinization modelling cannot be performed beyond this temperature without introducing large uncertainties.

To our knowledge, the Pitzer database is the largest and most consistent database that allows consideration of temperature and pressure variations in geochemical calculations at high ionic strength (>1.5M). Some aqueous ion pairs and complexes, in particular those related to the amphoteric behaviour of carbonates and sulfates, are included in the database and therefore in the model.

The Pitzer coefficients are one of the main potential sources of uncertainty in this thermodynamic approach. For the sensitivity tests, we compared the results obtained with the Pitzer database^50^ (distributed with PhreeqC) and those obtained with the PhreeSCALE code^55^ and its associated thermodynamic database at 25 °C (data are not available at higher temperatures in the database associated with PhreeSCALE)^8,55^ but found no considerable difference. Furthermore, elements such as aluminium, ferric iron or sulfur were not considered at either ambient temperature or temperatures above ambient temperature. Therefore, oxidation and reduction processes, as well as the behaviour of clay minerals, cannot be assessed. The possibility of studying salt formation by alteration of iron-rich peridotite, which is the reaction thought to be responsible for the production of hydrogen and magnetite (Fe_3_O_4_), is also disregarded. In addition, some phases are not available in the databases and thus cannot be included in the geochemical calculations, such as CaCl_2_MgCl_2_:12H_2_O and CaCl_2_MgCl_2_:6H_2_O, which should in practice replace tachyhydrite above 110 °C^53^. In summary, the major geochemical processes acting in the generation of highly concentrated brines by serpentinization can be described. Nevertheless, additional efforts are required to consistently extend the applicability of the thermodynamic approach to more complex and realistic systems.

### Calculation methodology

Salt formation is a response to water consumption by serpentinization. A script (calculation loops) was written especially for this purpose. Considering, for simplicity, the Mg end-member (enstatite) of orthopyroxene with a porosity of 10%, one kilogram of water is initially contained in a rock composed of 288 moles of enstatite. At each time step, a fixed quantity of enstatite (0.1 mole) is placed in contact with the pore water. Then, this mineral dissolves and forms serpentine, which consumes water. Serpentine has a higher molar volume than Mg-rich orthopyroxene; thus, the reaction leads to a reduction in the porosity. Therefore, at each time step, a volume balance is made between the remaining fluid quantity and the remaining porosity in the rock. If this balance indicates a water deficit, the free volume inside the porosity is filled with seawater. In contrast, any surplus is expelled out of the system. Notably, the volumetric expansion due to serpentinization may lead to pore clogging or fracture formation, and the latter can sustain the serpentinization process. However, these two mechanisms are not considered in our calculation. In addition, serpentinization of enstatite releases Si (equation 1), which, in turn, can form secondary products such as talc and quartz, two mineral phases that are observed in serpentine. At local thermodynamic equilibrium, the Gibbs phase rule does not permit the co-existence of these two phases; therefore, both mineral controls were tested separately. The results of considering talc in the calculations suggest that Mg cannot be entirely trapped in this phase during serpentinization and that a large amount of Mg remains in solution. On the contrary, serpentinization of olivine (e.g. forsterite) consumed Si (equation 2) that prevents the formation of talc and quartz but still allow the formation of salt (calculation not shown). In addition, this reaction consumes two times more water than the serpentinization of enstatite and can finally lead to the formation of a higher amount of salt at a similar alteration rate. However, as it prevents the precipitation of quartz and talc that are usually associated with serpentine^46^, we choose to focus on the orthopyroxene end member to investigate the peridotite serpentinization in this study.

2 $$3M{g}_{2}Si{O}_{4}(Olivine)+Si{O}_{2(aq)}+4{H}_{2}O=M{g}_{3}S{i}_{2}{O}_{5}{(OH)}_{4}$$

## Supplementary information

Supplementary information

## Data Availability

Data sets generated during the current study are available in Supplementary Information or from the corresponding author on reasonable request.
