# Supplementary material for: Thermodynamic evidence of giant salt deposit formation by serpentinization: an alternative mechanism to solar evaporation
Source: Sci Rep. 2019 Aug 12;9:11720. doi: 10.1038/s41598-019-48138-9 (PMC6690867; doi:10.1038/s41598-019-48138-9)
Supplement: Supplementary file 1 — Supplementary information [file 41598_2019_48138_MOESM1_ESM.docx]

Thermodynamic evidence of giant salt deposit formation by serpentinization: an alternative mechanism to solar evaporation.

Mathieu Debure^1,^^[[1]](#footnote-1)^, Arnault Lassin^1^, Nicolas Marty^1^, Francis Claret^1^, Aurélien Virgone^2^, Sylvain Calassou^2^, Eric C. Gaucher^2^

^1^ BRGM – French Geological Survey - 45060 Orléans - France.

^2^ TOTAL, CSTJF, Avenue Larribau, F-64018 Pau Cedex, France

# Supplementary information

The supplementary information presents all the results of the calculations performed. Supplementary Information 1 shows the mineralogical assemblage at the end of the calculations. Supplementary Information 2 shows the salt formation as a function of water consumption. Supplementary Information 3 gives the brine composition once the water is consumed by serpentinization. Supplementary Information 4 gives the salt composition once the hot brines are cooled by their interactions with seawater.

# **Supplementary information 1:** Serpentinization of Mg-rich orthopyroxene (enstatite) by seawater.

**Fig. S1. Serpentine (lizardite) and the formation of other secondary phases after Mg-rich orthopyroxene (enstatite) alteration with seawater as a function of CO_2_ partial pressure and fixed temperature (25 °C). a,** free CO_2_ partial pressure (here 10^-3.44^ MPa at the end of the reaction). **b,** CO_2_ partial pressure of 10^-3.44^ MPa. **c,** CO_2_ partial pressure 1.01325 MPa. **d,** CO_2_ partial pressure of 10.1325 MPa. Talc is discarded from the reaction.

**Fig. S2. Serpentine (lizardite) and the formation of other secondary phases after Mg-rich orthopyroxene (enstatite) alteration with seawater as a function of CO_2_ partial pressure and fixed temperature (25 °C). a,** free CO_2_ partial pressure (here 10^-3.75^ MPa at the end of the reaction). **b,** CO_2_ partial pressure of 10^-3.44^ MPa. **c,** CO_2_ partial pressure 1.01325 MPa. **d,** CO_2_ partial pressure of 10.1325 MPa. Talc is considered at thermodynamic equilibrium.

**Fig. S3. Serpentine (lizardite) and the formation of other secondary phases after Mg-rich orthopyroxene (enstatite) alteration with seawater as a function of CO_2_ partial pressure and fixed temperature (150 °C). a,** free CO_2_ partial pressure (here 17.7 MPa at the end of the reaction). **b,** CO_2_ partial pressure of 10^-3.44^ MPa. **c,** CO_2_ partial pressure 1.01325 MPa. **d,** CO_2_ partial pressure of 10.1325 MPa. Talc is discarded from the reaction.

**Fig. S4. Serpentine (lizardite) and the formation of other secondary phases after Mg-rich orthopyroxene (enstatite) alteration with seawater as a function of CO_2_ partial pressure and fixed temperature (150 °C). a,** free CO_2_ partial pressure (here 7.2 MPa at the end of the reaction). **b,** CO_2_ partial pressure of 10^-3.44^ MPa. **c,** CO_2_ partial pressure 1.01325 MPa. **d,** CO_2_ partial pressure of 10.1325 MPa. Talc is considered at thermodynamic equilibrium.

**Fig. S5. Serpentine (lizardite) and the formation of other secondary phases after Mg-rich orthopyroxene (enstatite) alteration with seawater as a function of CO_2_ partial pressure and fixed temperature (250 °C). a,** free CO_2_ partial pressure (here 114.5 MPa at the end of the reaction). **b,** CO_2_ partial pressure of 10^-3.44^ MPa. **c,** CO_2_ partial pressure 1.01325 MPa. **d,** CO_2_ partial pressure of 10.1325 MPa. Talc is discarded from the reaction.

**Fig. S6. Serpentine (lizardite) and the formation of other secondary phases after Mg-rich orthopyroxene (enstatite) alteration with seawater as a function of CO_2_ partial pressure and fixed temperature (250 °C). a,** free CO_2_ partial pressure (here 94 MPa at the end of the reaction). **b,** CO_2_ partial pressure of 10^-3.44^ MPa. **c,** CO_2_ partial pressure 1.01325 MPa. **d,** CO_2_ partial pressure of 10.1325 MPa. Talc is considered at thermodynamic equilibrium.

# **Supplementary information 2:** Salt evolution as a function of the consumption of water by serpentinization.

**Carbonates as a function of the water content:** carbonates are observed at 25°C and 150°C but not at 250°C.


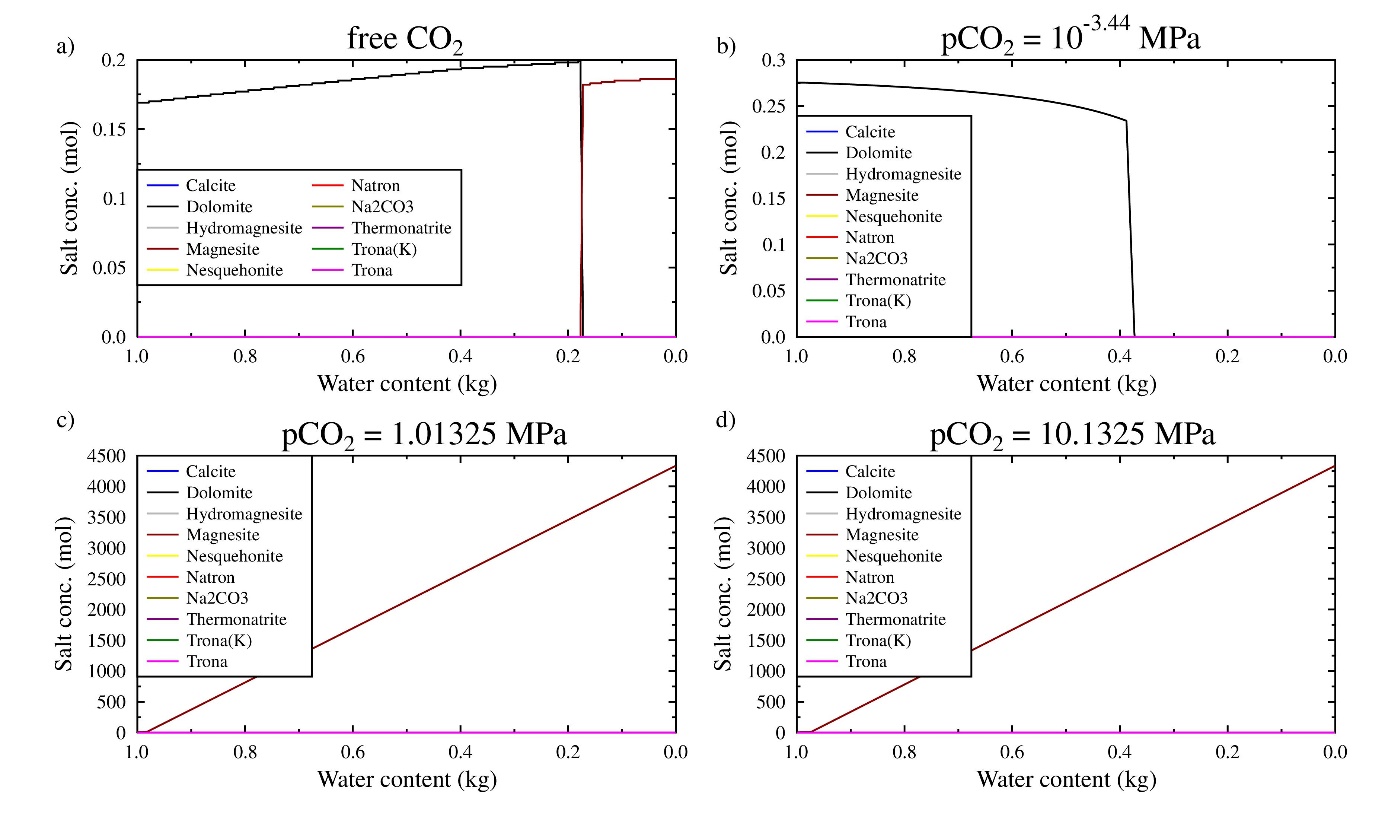


**Fig. S7. Secondary carbonates formed by serpentinization as a function of CO_2_ partial pressure and fixed temperature (25 °C). a,** free CO_2_ partial pressure (here 10^-3.44^ MPa at the end of the reaction). **b,** CO_2_ partial pressure of 10^-3.44^ MPa. **c,** CO_2_ partial pressure 1.01325 MPa. **d,** CO_2_ partial pressure of 10.1325 MPa. Only dolomite and magnesite forms.


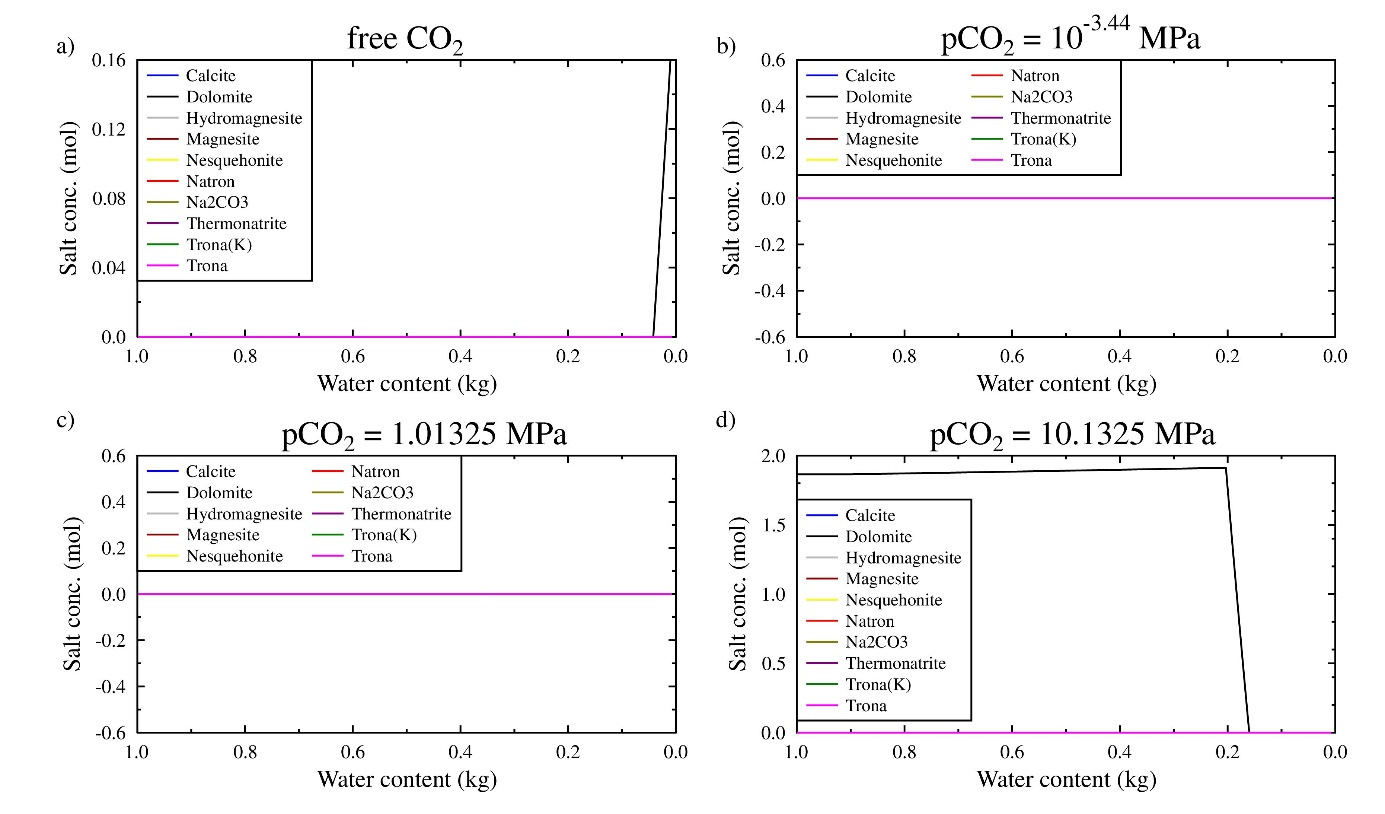


**Fig. S8. Secondary carbonates formed by serpentinization as a function of CO_2_ partial pressure and fixed temperature (150 °C). a,** free CO_2_ partial pressure (here 17.7 MPa). **b,** CO_2_ partial pressure of 10^-3.44^ MPa. **c,** CO_2_ partial pressure 1.01325 MPa. **d,** CO_2_ partial pressure of 10.1325 MPa. Only dolomite forms.

**Chlorines as a function of the water content:**


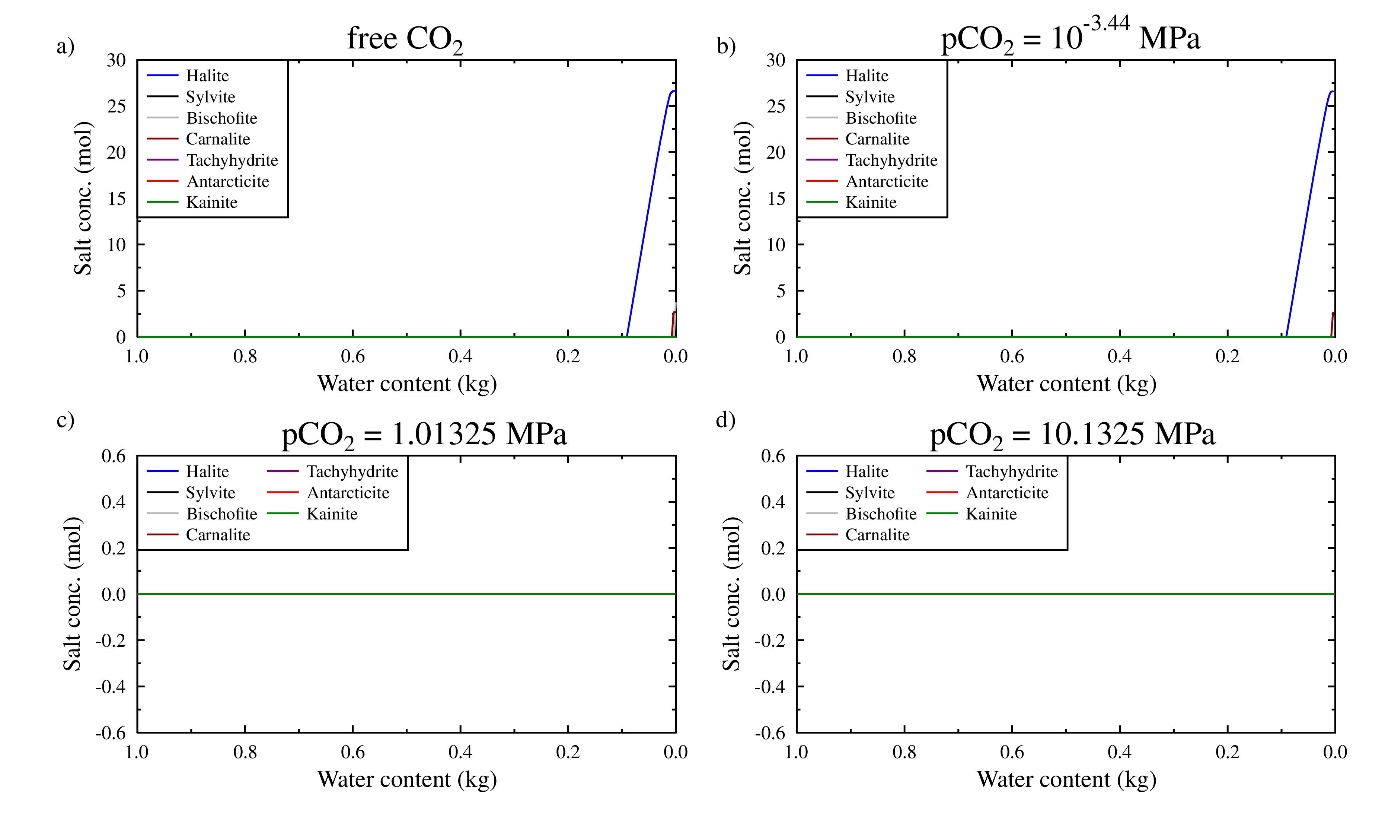


**Fig. S9. Secondary chlorines formed by serpentinization as a function of CO_2_ partial pressure and fixed temperature (25 °C). a,** free CO_2_ partial pressure (here 10^-3.44^ MPa at the end of the reaction). **b,** CO_2_ partial pressure of 10^-3.44^ MPa. **c,** CO_2_ partial pressure 1.01325 MPa. **d,** CO_2_ partial pressure of 10.1325 MPa. Only halite and carnallite (free CO_2_) forms.


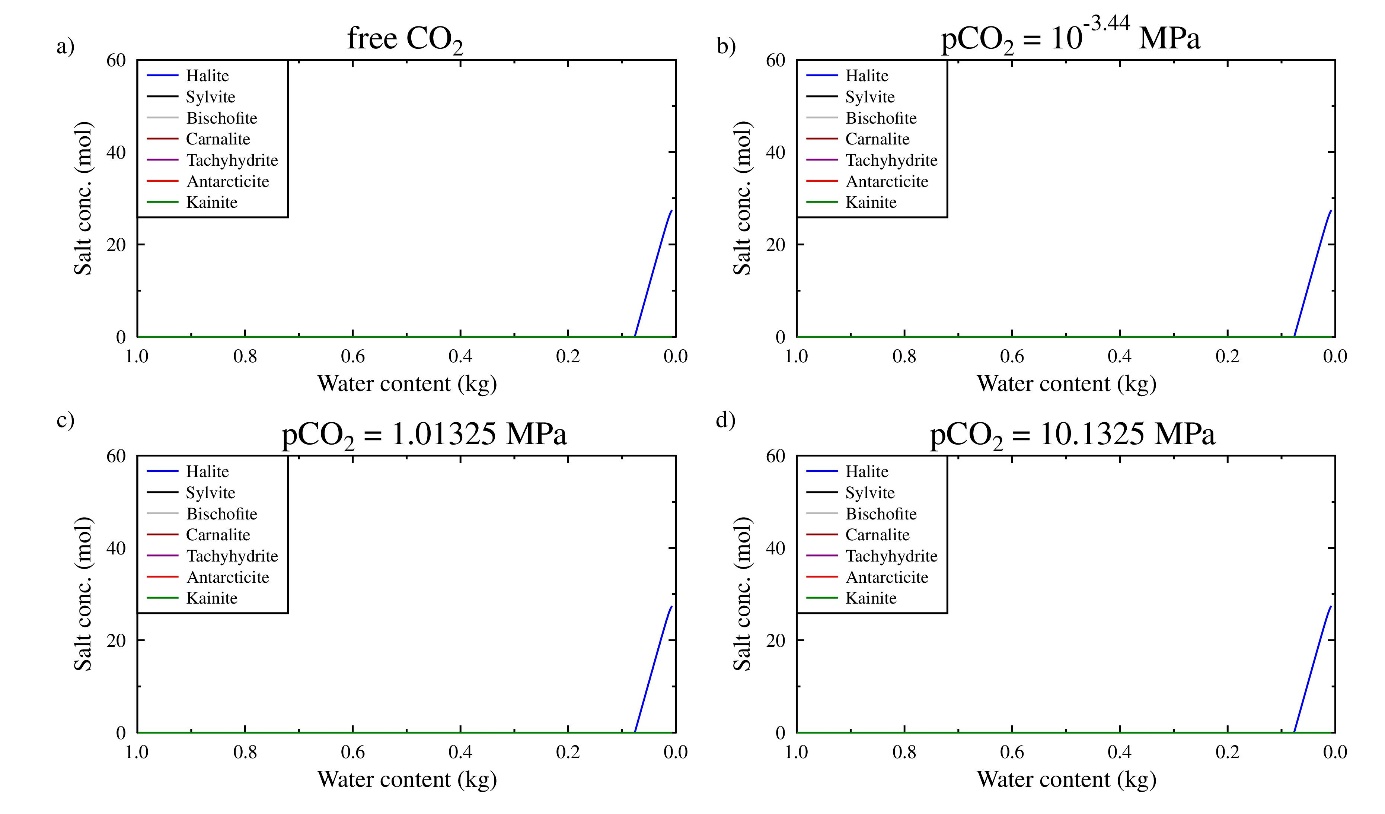


**Fig. S10. Secondary chlorines formed by serpentinization as a function of CO_2_ partial pressure and fixed temperature (150 °C). a,** free CO_2_ partial pressure (here 17.7 MPa at the end of the reaction). **b,** CO_2_ partial pressure of 10^-3.44^ MPa. **c,** CO_2_ partial pressure 1.01325 MPa. **d,** CO_2_ partial pressure of 10.1325 MPa. Only halite forms.


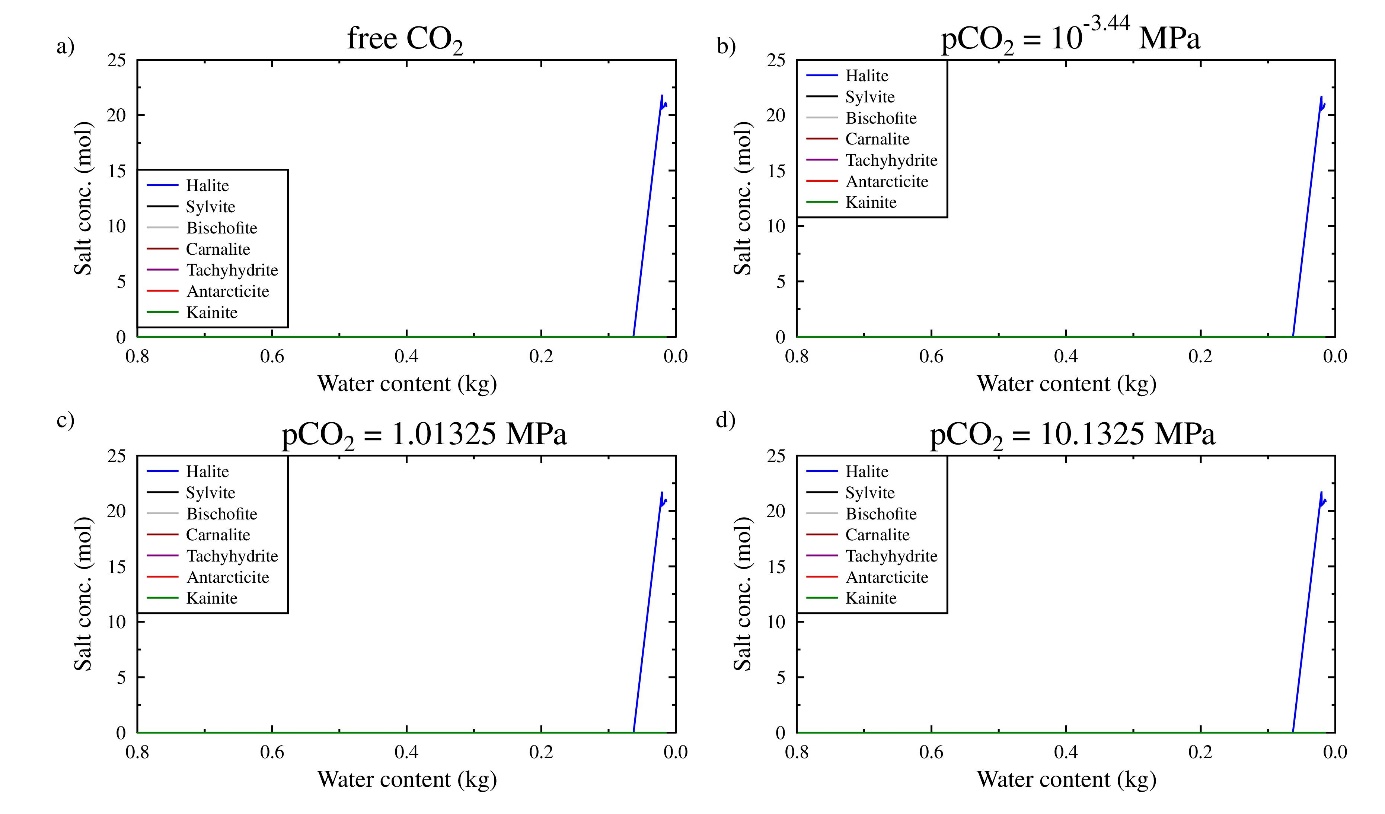


**Fig. S11. Secondary chlorines formed by serpentinization as a function of CO_2_ partial pressure and fixed temperature (250 °C). a,** free CO_2_ partial pressure (here 114.5 MPa at the end of the reaction). **b,** CO_2_ partial pressure of 10^-3.44^ MPa. **c,** CO_2_ partial pressure 1.1325 MPa. **d,** CO_2_ partial pressure of 10.1325 MPa. Only halite forms.

**Sulfates as a function of the water content:**


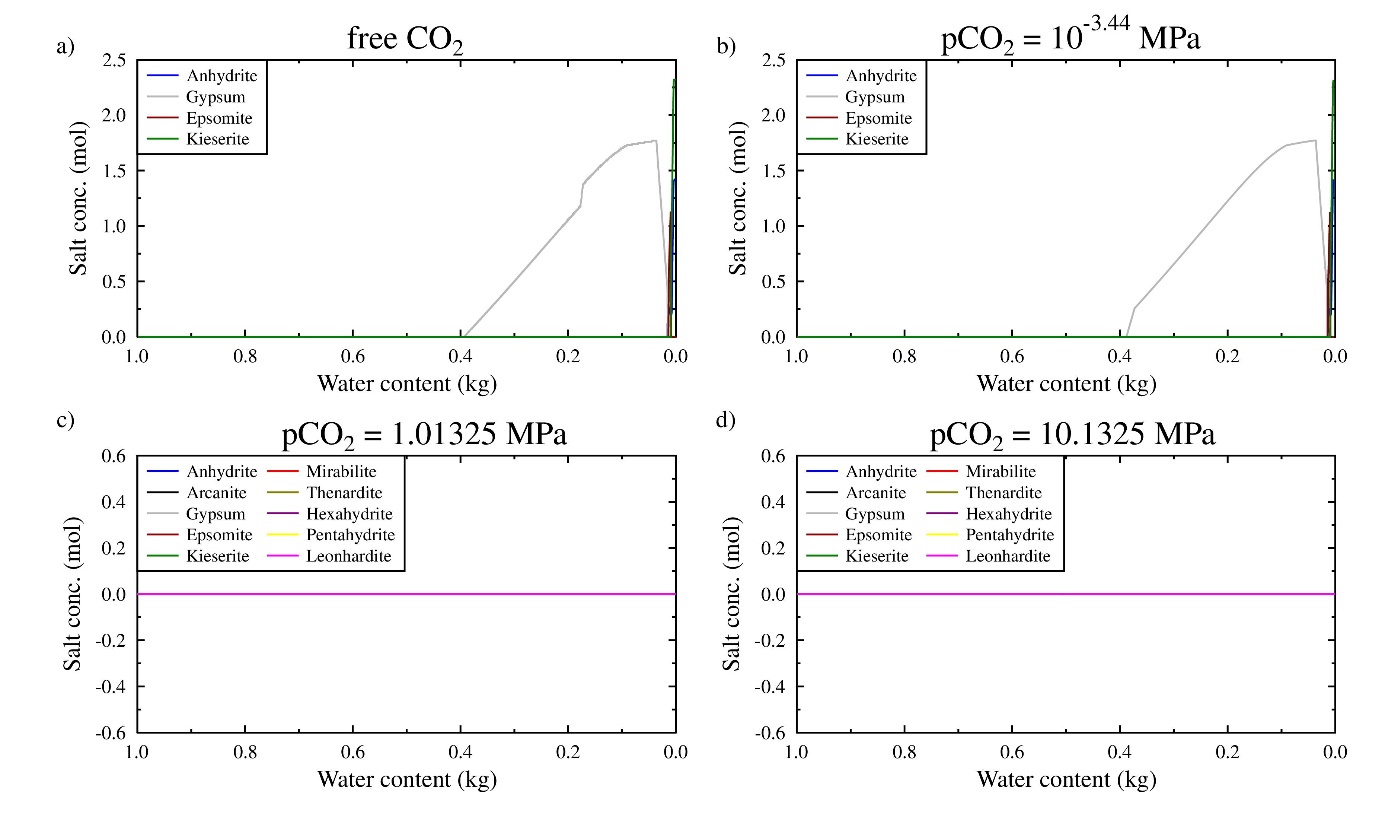


**Fig. S12. Secondary sulfates formed by serpentinization as a function of CO_2_ partial pressure and fixed temperature (25 °C). a,** free CO_2_ partial pressure (here 10^-3.44^ MPa at the end of the reaction); gypsum, anhydrite, epsomite and kieserite forms. **b,** CO_2_ partial pressure of 10^-3.44^ MPa; gypsum, anhydrite and epsomite forms. **c,** CO_2_ partial pressure 1.01325 MPa. **d,** CO_2_ partial pressure of 10.1325 MPa.


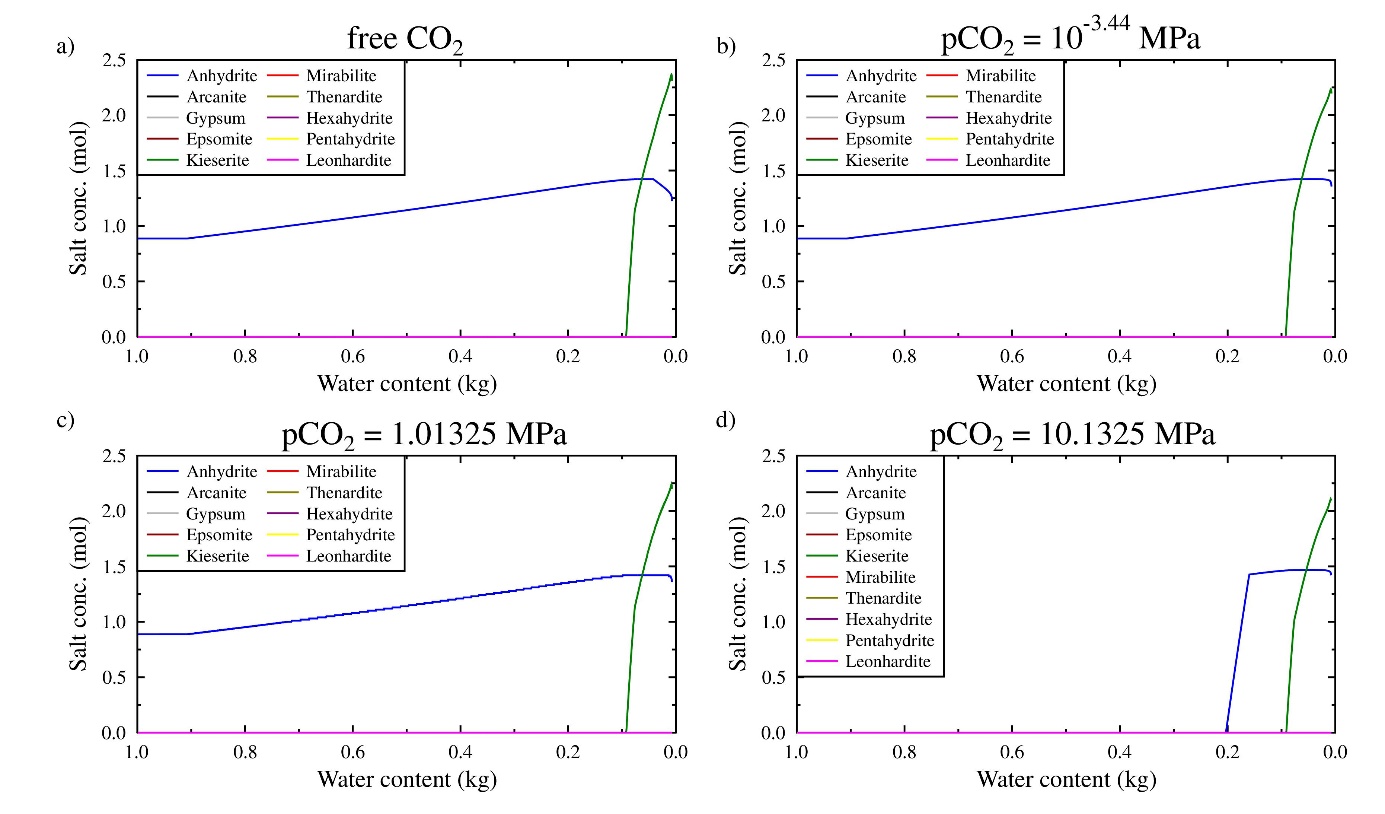


**Fig. S13. Secondary sulfates formed by serpentinization as a function of CO_2_ partial pressure and fixed temperature (150 °C). a,** free CO_2_ partial pressure (here 17.7 MPa at the end of the reaction). **b,** CO_2_ partial pressure of 10^-3.44^ MPa. **c,** CO_2_ partial pressure 1.01325 MPa. **d,** CO_2_ partial pressure of 10.1325 MPa. Only halite and kieserite forms.


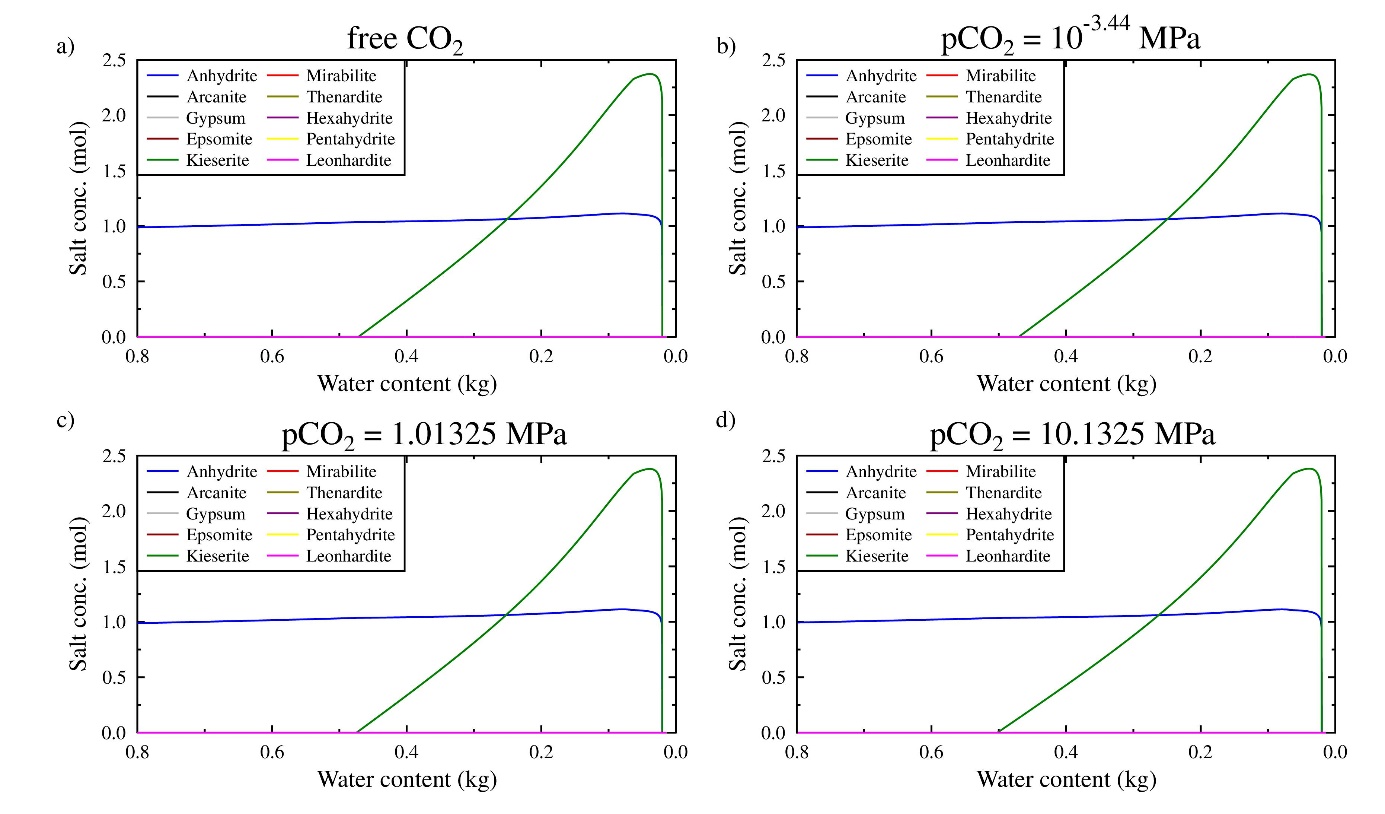


**Fig. S14. Secondary sulfates formed by serpentinization as a function of CO_2_ partial pressure and fixed temperature (250 °C). a,** free CO_2_ partial pressure (here 114.5 MPa at the end of the reaction). **b,** CO_2_ partial pressure of 10^-3.44^ MPa. **c,** CO_2_ partial pressure 1.01325 MPa. **d,** CO_2_ partial pressure of 10.1325 MPa. Only halite and kieserite form.

**Complex salts as a function of the water content:** complex salts are observed at 25°C but not at 150°C and 250°C.


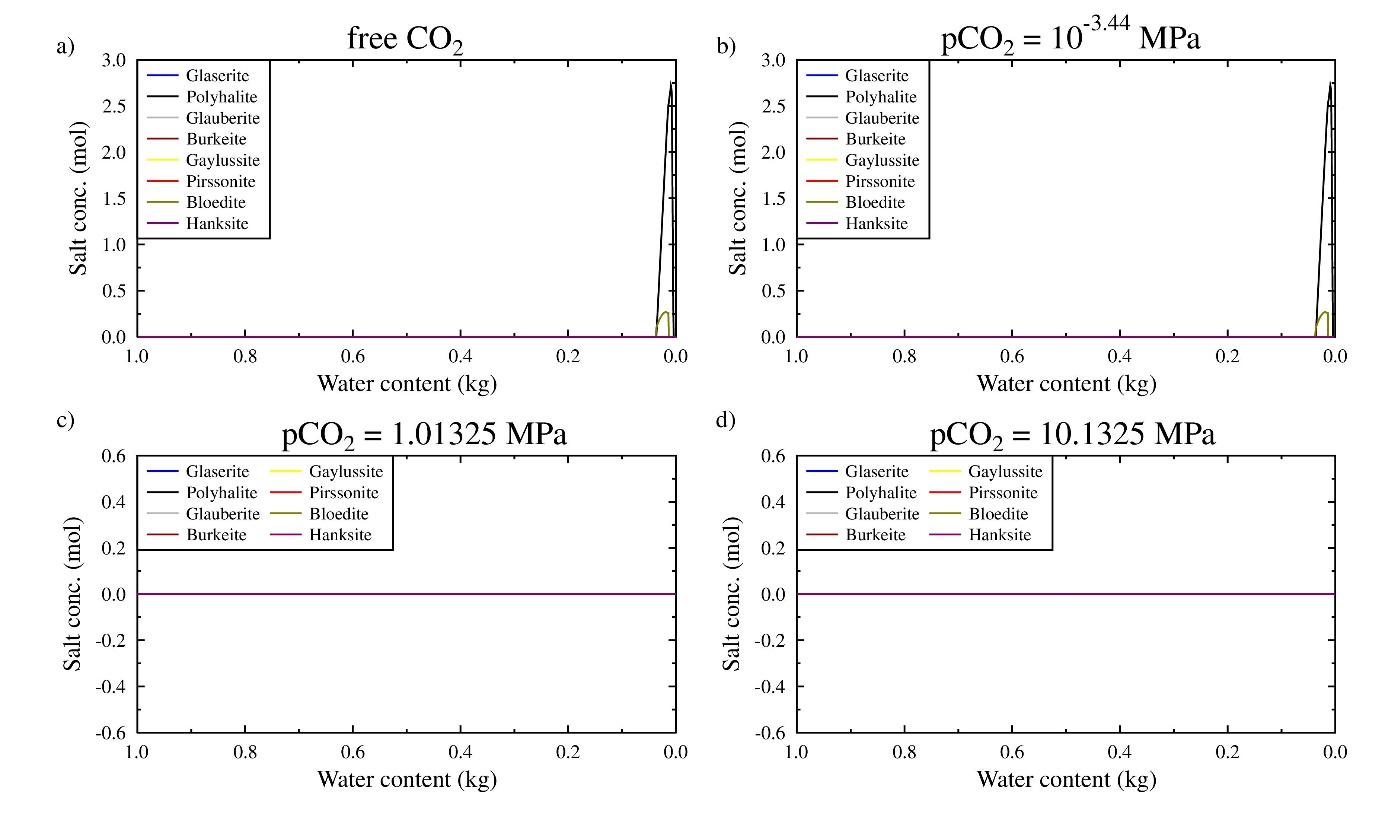


**Fig. S15. Secondary complex salt formed by serpentinization as a function of CO_2_ partial pressure and fixed temperature (25 °C). a,** free CO_2_ partial pressure (here 10^-3.44^ MPa at the end of the reaction). **b,** CO_2_ partial pressure of 10^-3.44^ MPa. **c,** CO_2_ partial pressure 1.01325 MPa. **d,** CO_2_ partial pressure of 10.1325 MPa. Only polyhalite and bloedite forms.

# **Supplementary information 3:** Brine composition after the peridotite alteration by seawater.

**Table S1: Brine composition (mol L^-1^) once the maximum amount of seawater is consumed by serpentinization.** The free pCO2 is equal to 10^-3.44^ MPa at 25°C, 17.7 MPa at 150°C and 114.5 MPa at 250°C.

| pCO_2_ | free | atm | 1 atm* | 10 atm | free | atm | 1 atm | 10 atm | free | atm | 1 atm | 10 atm |
| --- | --- | --- | --- | --- | --- | --- | --- | --- | --- | --- | --- | --- |
| Temperature | 25 | 25 | 25 | 25 | 150 | 150 | 150 | 150 | 250 | 250 | 250 | 250 |
| pH | 5.93E+00 | 5.93E+00 | 6.05E+00 | 5.50E+00 | 4.42E+00 | 4.42E+00 | 4.42E+00 | 4.44E+00 | 4.25E+00 | 4.26E+00 | 4.25E+00 | 4.25E+00 |
| Alkalinity | 3.00E-04 | 3.00E-04 | 2.57E-02 | 6.97E-02 | 1.66E-02 | 5.45E-04 | 1.41E-03 | 7.09E-03 | 5.03E-02 | 9.12E-04 | 1.46E-03 | 5.05E-03 |
| Ionic strength | 1.76E+01 | 1.76E+01 | 7.58E-01 | 8.24E-01 | 1.82E+01 | 1.80E+01 | 1.80E+01 | 1.72E+01 | 2.10E+01 | 1.96E+01 | 2.07E+01 | 2.08E+01 |
| Remaining H_2_O (%) | 2.98E-04 | 3.39E-03 | 9.53E-04 | 6.01E-04 | 7.10E-03 | 7.14E-03 | 7.13E-03 | 7.46E-03 | 1.41E-02 | 1.49E-02 | 1.42E-02 | 1.42E-02 |
| Electrical balance error | 3.03E-07 | 1.60E-08 | 2.41E-09 | 1.23E-09 | 1.89E-09 | 3.18E-09 | 1.67E-09 | 9.16E-10 | 5.30E-11 | 3.86E-10 | 7.93E-11 | 1.78E-10 |
| Si | 1.15E-06 | 1.15E-06 | 1.60E-04 | 1.57E-04 | 1.84E-04 | 1.88E-04 | 1.87E-04 | 2.09E-04 | 4.27E-03 | 4.04E-03 | 4.25E-03 | 4.25E-03 |
| Na | 5.00E-02 | 5.00E-02 | 4.86E-01 | 4.86E-01 | 5.06E-01 | 5.14E-01 | 5.13E-01 | 5.90E-01 | 7.23E+00 | 6.56E+00 | 7.09E+00 | 7.11E+00 |
| Ca | 1.81E-03 | 1.81E-03 | 2.61E-03 | 3.11E-03 | 8.82E-02 | 8.25E-02 | 8.27E-02 | 6.08E-02 | 5.45E-01 | 5.20E-01 | 5.42E-01 | 5.42E-01 |
| K | 1.08E-02 | 1.08E-02 | 1.06E-02 | 1.06E-02 | 1.45E+00 | 1.44E+00 | 1.44E+00 | 1.38E+00 | 6.39E-01 | 6.09E-01 | 6.35E-01 | 6.36E-01 |
| Mg | 5.83E+00 | 5.83E+00 | 7.48E-02 | 9.63E-02 | 5.18E+00 | 5.13E+00 | 5.13E+00 | 4.90E+00 | 3.28E+00 | 3.10E+00 | 3.24E+00 | 3.25E+00 |
| Cl | 1.17E+01 | 1.17E+01 | 5.67E-01 | 5.67E-01 | 1.16E+01 | 1.16E+01 | 1.16E+01 | 1.12E+01 | 1.21E+01 | 1.12E+01 | 1.20E+01 | 1.20E+01 |
| SO_4_^2-^ | 1.25E-02 | 1.25E-02 | 2.93E-02 | 2.93E-02 | 4.24E-01 | 3.98E-01 | 3.99E-01 | 3.40E-01 | 1.67E+00 | 1.59E+00 | 1.66E+00 | 1.67E+00 |
| HCO_3_^-^ | 5.42E-05 | 5.42E-05 | 5.60E-02 | 3.58E-01 | 3.39E-02 | 6.78E-07 | 1.91E-03 | 1.79E-02 | 1.34E-01 | 4.03E-07 | 1.16E-03 | 1.15E-02 |
| Salinity (psu) | 186 | 186 | 56 | 75 | 283 | 276 | 277 | 265 | 506 | 467 | 492 | 494 |

* 1 atm = 1.01325 MPa

**Table S2: Brine composition (mol L^-1^) once the 99.0 % of seawater is consumed by serpentinization.** The free pCO2 is equal to 10^-3.23^ MPa at 25°C, 18.3 MPa at 150°C and 114.5 MPa at 250°C.

| pCO_2_ | free | atm | 1 atm* | 10 atm | free | atm | 1 atm | 10 atm | free | atm | 1 atm | 10 atm |
| --- | --- | --- | --- | --- | --- | --- | --- | --- | --- | --- | --- | --- |
| Temperature | 25 | 25 | 25 | 25 | 150 | 150 | 150 | 150 | 250 | 250 | 250 | 250 |
| pH | 6.52E+00 | 6.52E+00 | 6.05E+00 | 5.50E+00 | 4.57E+00 | 4.57E+00 | 4.57E+00 | 4.57E+00 | 4.25E+00 | 4.26E+00 | 4.25E+00 | 4.25E+00 |
| Alkalinity | 1.36E-04 | 1.05E-04 | 2.57E-02 | 6.97E-02 | 4.25E-03 | 2.79E-04 | 4.98E-04 | 2.41E-03 | 5.03E-02 | 9.12E-04 | 1.46E-03 | 5.05E-03 |
| Ionic strength | 1.11E+01 | 1.11E+01 | 7.58E-01 | 8.24E-01 | 1.13E+01 | 1.13E+01 | 1.13E+01 | 1.12E+01 | 2.10E+01 | 1.96E+01 | 2.07E+01 | 2.08E+01 |
| Remaining H_2_O (%) | 1.40E-02 | 1.40E-02 | 1.44E-02 | 1.39E-02 | 1.41E-02 | 1.41E-02 | 1.41E-02 | 1.44E-02 | 1.41E-02 | 1.49E-02 | 1.42E-02 | 1.42E-02 |
| Electrical balance error | 1.07E-08 | 6.38E-09 | 2.30E-09 | 1.20E-09 | 1.40E-09 | 2.34E-09 | 1.22E-09 | 6.58E-10 | 5.30E-11 | 3.86E-10 | 7.93E-11 | 1.78E-10 |
| Si | 1.69E-05 | 1.70E-05 | 1.60E-04 | 1.57E-04 | 5.20E-04 | 5.22E-04 | 5.22E-04 | 5.30E-04 | 4.27E-03 | 4.04E-03 | 4.25E-03 | 4.25E-03 |
| Na | 1.22E+00 | 1.22E+00 | 4.86E-01 | 4.86E-01 | 2.49E+00 | 2.51E+00 | 2.51E+00 | 2.58E+00 | 7.23E+00 | 6.56E+00 | 7.09E+00 | 7.11E+00 |
| Ca | 2.32E-03 | 2.32E-03 | 2.61E-03 | 3.11E-03 | 1.08E-02 | 1.08E-02 | 1.08E-02 | 1.04E-02 | 5.45E-01 | 5.20E-01 | 5.42E-01 | 5.42E-01 |
| K | 9.69E-02 | 9.71E-02 | 1.06E-02 | 1.06E-02 | 7.33E-01 | 7.31E-01 | 7.31E-01 | 7.16E-01 | 6.39E-01 | 6.09E-01 | 6.35E-01 | 6.36E-01 |
| Mg | 3.11E+00 | 3.10E+00 | 7.48E-02 | 9.63E-02 | 2.63E+00 | 2.62E+00 | 2.62E+00 | 2.57E+00 | 3.28E+00 | 3.10E+00 | 3.24E+00 | 3.25E+00 |
| Cl | 6.52E+00 | 6.52E+00 | 5.67E-01 | 5.67E-01 | 8.11E+00 | 8.11E+00 | 8.11E+00 | 8.07E+00 | 1.21E+01 | 1.12E+01 | 1.20E+01 | 1.20E+01 |
| SO_4_^2-^ | 5.04E-01 | 5.04E-01 | 2.93E-02 | 2.93E-02 | 1.92E-01 | 1.95E-01 | 1.95E-01 | 1.91E-01 | 1.67E+00 | 1.59E+00 | 1.66E+00 | 1.67E+00 |
| HCO_3_^-^ | 7.10E-05 | 4.40E-05 | 5.60E-02 | 3.58E-01 | 4.18E-02 | 8.11E-07 | 2.28E-03 | 2.27E-02 | 1.34E-01 | 4.03E-07 | 1.16E-03 | 1.15E-02 |
| Salinity (psu) | 195 | 195 | 56 | 75 | 212 | 210 | 210 | 211 | 506 | 467 | 492 | 494 |

* 1 atm = 1.01325 MPa

**Table S3: Brine composition (mol L^-1^) once the maximum amount of seawater is consumed by serpentinization and talc precipitated.** The free pCO2 is equal to 10^-3.75^ MPa at 25°C, 7.2 MPa at 150°C and 94 MPa at 250°C.

| pCO_2_ | free | atm | 1 atm* | 10 atm | free | atm | 1 atm | 10 atm | free | atm | 1 atm | 10 atm |
| --- | --- | --- | --- | --- | --- | --- | --- | --- | --- | --- | --- | --- |
| Temperature | 25 | 25 | 25 | 25 | 150 | 150 | 150 | 150 | 250 | 250 | 250 | 250 |
| pH | 6.08E+00 | 6.61E+00 | 6.05E+00 | 5.50E+00 | 4.62E+00 | 4.62E+00 | 4.62E+00 | 4.61E+00 | 4.45E+00 | 4.41E+00 | 4.43E+00 | 4.45E+00 |
| Alkalinity | 3.88E-04 | 1.40E-04 | 2.57E-02 | 6.97E-02 | 1.18E-02 | 8.69E-04 | 2.34E-03 | 1.45E-02 | 6.34E-02 | 2.38E-03 | 2.80E-03 | 7.69E-03 |
| Ionic strength | 1.76E+01 | 1.12E+01 | 7.58E-01 | 8.24E-01 | 1.84E+01 | 1.82E+01 | 1.83E+01 | 1.80E+01 | 2.09E+01 | 2.21E+01 | 2.14E+01 | 2.07E+01 |
| Remaining H_2_O (%) | 7.80E-04 | 3.36E-03 | 9.53E-04 | 6.01E-04 | 6.85E-03 | 6.87E-03 | 6.87E-03 | 6.89E-03 | 1.38E-02 | 1.34E-02 | 1.37E-02 | 1.39E-02 |
| Electrical balance error | 1.10E-07 | 1.44E-09 | 7.08E-09 | 3.12E-10 | 7.68E-08 | 8.72E-08 | 9.23E-08 | -3.49E-10 | 1.11E-10 | 3.87E-10 | 3.73E-10 | 6.60E-10 |
| Si | 3.99E-07 | 8.06E-06 | 1.60E-04 | 1.57E-04 | 4.67E-05 | 4.72E-05 | 4.72E-05 | 4.76E-05 | 1.04E-03 | 1.13E-03 | 1.08E-03 | 1.03E-03 |
| Na | 5.00E-02 | 1.16E+00 | 4.86E-01 | 4.86E-01 | 4.94E-01 | 4.97E-01 | 4.97E-01 | 4.94E-01 | 7.31E+00 | 8.21E+00 | 7.64E+00 | 7.15E+00 |
| Ca | 1.81E-03 | 2.25E-03 | 2.61E-03 | 3.11E-03 | 9.76E-02 | 9.14E-02 | 9.17E-02 | 3.81E-02 | 5.49E-01 | 5.65E-01 | 5.58E-01 | 5.47E-01 |
| K | 1.08E-02 | 9.52E-02 | 1.06E-02 | 1.06E-02 | 1.46E+00 | 1.46E+00 | 1.46E+00 | 1.46E+00 | 6.28E-01 | 6.47E-01 | 6.40E-01 | 6.27E-01 |
| Mg | 5.83E+00 | 3.16E+00 | 7.48E-02 | 9.63E-02 | 5.24E+00 | 5.20E+00 | 5.20E+00 | 5.18E+00 | 3.23E+00 | 3.29E+00 | 3.27E+00 | 3.20E+00 |
| Cl | 1.17E+01 | 6.59E+00 | 5.67E-01 | 5.67E-01 | 1.17E+01 | 1.17E+01 | 1.17E+01 | 1.17E+01 | 1.21E+01 | 1.32E+01 | 1.25E+01 | 1.20E+01 |
| SO_4_^2-^ | 1.25E-02 | 4.94E-01 | 2.93E-02 | 2.93E-02 | 4.49E-01 | 4.23E-01 | 4.25E-01 | 3.48E-01 | 1.65E+00 | 1.70E+00 | 1.69E+00 | 1.65E+00 |
| HCO_3_^-^ | 4.14E-05 | 5.73E-05 | 5.60E-02 | 3.58E-01 | 1.80E-02 | 8.82E-07 | 2.49E-03 | 2.41E-02 | 1.31E-01 | 5.87E-07 | 1.49E-03 | 1.36E-02 |
| Salinity (psu) | 186 | 194 | 56 | 75 | 286 | 281 | 281 | 273 | 504 | 526 | 509 | 492 |

* 1 atm = 1.01325 MPa

**Table S4: Brine composition (mol L^-1^) once the 99.0 % of seawater is consumed by serpentinization and talc precipitated.** The free pCO2 is equal to 10^-3.43^ MPa at 25°C, 8.2 MPa at 150°C and 94 MPa at 250°C.

| pCO_2_ | free | atm | 1 atm* | 10 atm | free | atm | 1 atm | 10 atm | free | atm | 1 atm | 10 atm |
| --- | --- | --- | --- | --- | --- | --- | --- | --- | --- | --- | --- | --- |
| Temperature | 25 | 25 | 25 | 25 | 150 | 150 | 150 | 150 | 250 | 250 | 250 | 250 |
| pH | 6.63E+00 | 6.63E+00 | 6.05E+00 | 5.50E+00 | 4.74E+00 | 4.74E+00 | 4.74E+00 | 4.74E+00 | 4.45E+00 | 4.41E+00 | 4.43E+00 | 4.45E+00 |
| Alkalinity | 1.37E-04 | 1.36E-04 | 2.57E-02 | 6.97E-02 | 3.19E-03 | 4.78E-04 | 8.12E-04 | 3.77E-03 | 6.34E-02 | 2.38E-03 | 2.80E-03 | 7.69E-03 |
| Ionic strength | 1.09E+01 | 1.08E+01 | 7.58E-01 | 8.24E-01 | 1.16E+01 | 1.16E+01 | 1.16E+01 | 1.15E+01 | 2.09E+01 | 2.21E+01 | 2.14E+01 | 2.07E+01 |
| Remaining H_2_O (%) | 1.33E-02 | 1.33E-02 | 1.34E-02 | 1.29E-02 | 1.31E-02 | 1.31E-02 | 1.31E-02 | 1.31E-02 | 1.38E-02 | 1.34E-02 | 1.37E-02 | 1.39E-02 |
| Electrical balance error | 1.14E-08 | 1.34E-09 | 6.98E-09 | 2.92E-10 | 5.88E-08 | 6.64E-08 | 7.03E-08 | -2.81E-10 | 1.11E-10 | 3.87E-10 | 3.73E-10 | 6.60E-10 |
| Si | 9.15E-06 | 9.19E-06 | 1.60E-04 | 1.57E-04 | 1.49E-04 | 1.50E-04 | 1.50E-04 | 1.50E-04 | 1.04E-03 | 1.13E-03 | 1.08E-03 | 1.03E-03 |
| Na | 1.38E+00 | 1.38E+00 | 4.86E-01 | 4.86E-01 | 2.35E+00 | 2.36E+00 | 2.36E+00 | 2.35E+00 | 7.31E+00 | 8.21E+00 | 7.64E+00 | 7.15E+00 |
| Ca | 2.55E-03 | 2.56E-03 | 2.61E-03 | 3.11E-03 | 1.17E-02 | 1.17E-02 | 1.17E-02 | 7.89E-03 | 5.49E-01 | 5.65E-01 | 5.58E-01 | 5.47E-01 |
| K | 1.07E-01 | 1.07E-01 | 1.06E-02 | 1.06E-02 | 7.65E-01 | 7.63E-01 | 7.63E-01 | 7.64E-01 | 6.28E-01 | 6.47E-01 | 6.40E-01 | 6.27E-01 |
| Mg | 2.96E+00 | 2.95E+00 | 7.48E-02 | 9.63E-02 | 2.74E+00 | 2.73E+00 | 2.73E+00 | 2.74E+00 | 3.23E+00 | 3.29E+00 | 3.27E+00 | 3.20E+00 |
| Cl | 6.41E+00 | 6.41E+00 | 5.67E-01 | 5.67E-01 | 8.21E+00 | 8.21E+00 | 8.21E+00 | 8.21E+00 | 1.21E+01 | 1.32E+01 | 1.25E+01 | 1.20E+01 |
| SO_4_^2-^ | 4.94E-01 | 4.93E-01 | 2.93E-02 | 2.93E-02 | 1.99E-01 | 2.00E-01 | 1.99E-01 | 1.96E-01 | 1.65E+00 | 1.70E+00 | 1.69E+00 | 1.65E+00 |
| HCO_3_^-^ | 5.93E-05 | 5.80E-05 | 5.60E-02 | 3.58E-01 | 1.92E-02 | 8.39E-07 | 2.36E-03 | 2.33E-02 | 1.31E-01 | 5.87E-07 | 1.49E-03 | 1.36E-02 |
| Salinity (psu) | 194 | 194 | 56 | 75 | 212 | 211 | 211 | 212 | 504 | 526 | 509 | 492 |

* 1 atm = 1.01325 MPa


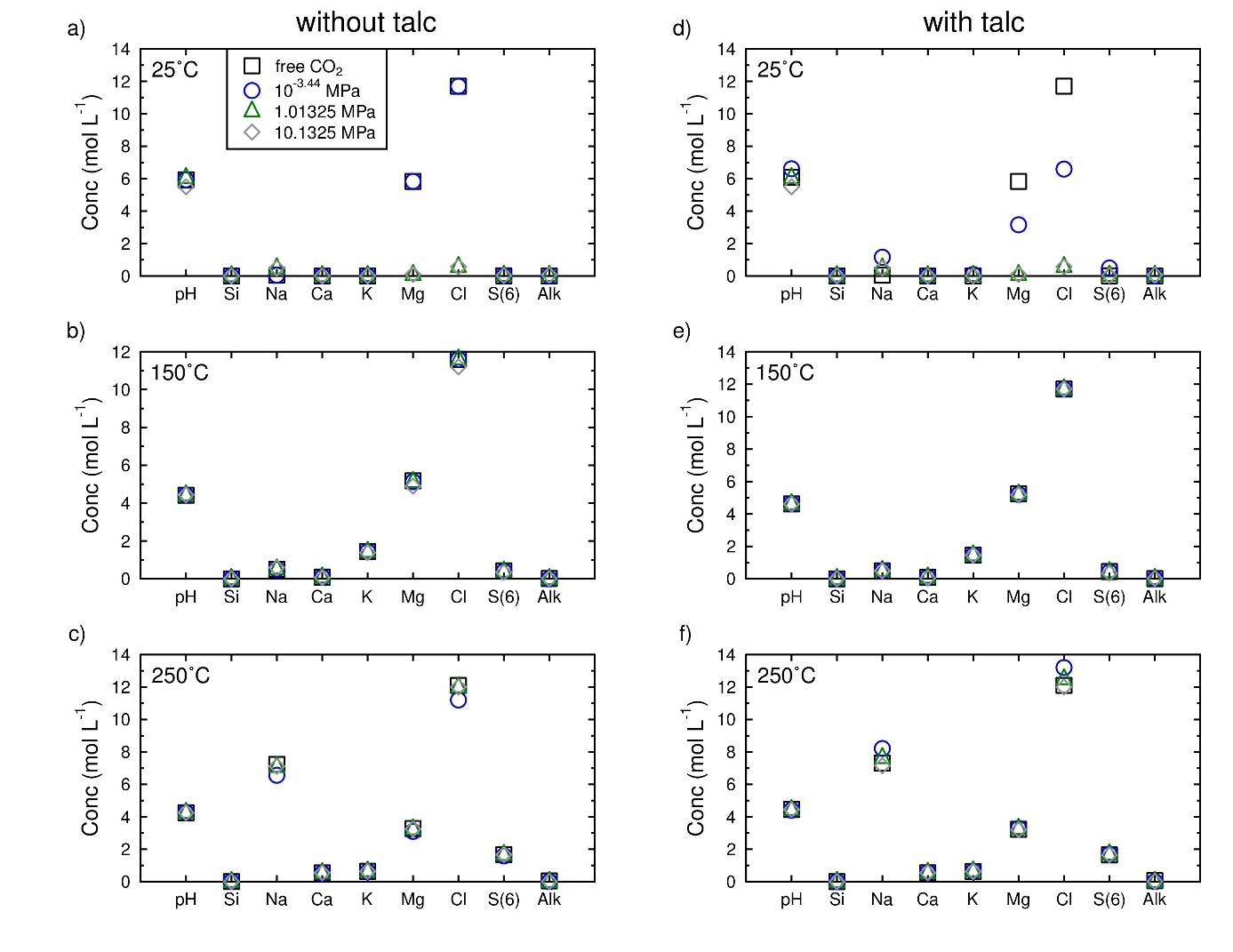


**Fig. S16. Brine composition once the seawater is consumed by serpentinization.** **a,** at 25°C without talc with 99.9% of the water consumed and free pCO_2_ equal to 10^-3.44^ MPa. **b,** at 150°C without talc with 99.5% of the water consumed and free pCO_2_ equal to 17.7 MPa. **c,** at 250°C without talc with 99.0% of the water consumed and free pCO_2_ equal to 114.5 MPa. **d,** at 25°C with talc with 99.9% of the water consumed and free pCO_2_ equal to 10^-3.75^ MPa. **e,** at 150°C with talc with 99.5% of the water consumed and free pCO_2_ equal to 7.2 MPa. **f,** at 250°C with talc with 99.0% of the water consumed and free pCO_2_ equal to 94 MPa.. The salinity range varies from 55 to 200 psu at 25 °C, 260 to 280 psu at 150°C and 470 to 530 psu at 250°C.

# **Supplementary information 4:** Composition of the salt precipitated when the seawater cools brines at the temperature representative of the bottom of the seas (4°C).

**Fig. S17. Additional salt precipitation after cooling the brines initially at 25°C to a temperature of 4°C representative of the bottom of the seas. a,** free CO_2_ partial pressure (here 10^-3.44^ MPa at the end of the reaction). **b,** CO_2_ partial pressure of 10^-3.44^ MPa. Salt does not precipitate at CO_2_ partial pressure of 1.01325 and 10.1325 MPa. The amount of minerals formed is given in wt%.

**Fig. S18. Additional salt precipitation after cooling the brines initially at 25°C to a temperature of 4°C representative of the bottom of the seas. a,** free CO_2_ partial pressure (here 10^-3.75^ MPa at the end of the reaction). **b,** CO_2_ partial pressure of 10^-3.44^ MPa. Salt does not precipitate at CO_2_ partial pressure of 1.01325 and 10.1325 MPa. The amount of minerals formed is given in wt%. Talc is considered at thermodynamic equilibrium.

**Fig. S19. Additional salt precipitation after cooling the brines initially at 150°C to a temperature of 4°C representative of the bottom of the seas. a,** free CO_2_ partial pressure (here 17.7 MPa at the end of the reaction). **b,** CO_2_ partial pressure of 10^-3.44^ MPa. **c,** CO_2_ partial pressure 1. 01325 MPa. **d,** CO_2_ partial pressure of 10.1325 MPa. The amount of minerals formed is given in wt%.

**Fig. S20. Additional salt precipitation after cooling the brines initially at 150°C to a temperature of 4°C representative of the bottom of the seas. a,** free CO_2_ partial pressure (here 7.2 MPa at the end of the reaction). **b,** CO_2_ partial pressure of 10^-3.44^ MPa. **c,** CO_2_ partial pressure 1.01325 MPa. **d,** CO_2_ partial pressure of 10.1325 MPa. The amount of minerals formed is given in wt%. Talc is considered at thermodynamic equilibrium.

**Fig. S21. Additional salt precipitation after cooling the brines initially at 250°C to a temperature of 4°C representative of the bottom of the seas. a,** free CO_2_ partial pressure (here 114.5 MPa at the end of the reaction). **b,** CO_2_ partial pressure of 10^-3.44^ MPa. **c,** CO_2_ partial pressure 1.01325 MPa. **d,** CO_2_ partial pressure of 10.1325 MPa. The amount of minerals formed is given in wt%.

**Fig. S22. Additional salt precipitation after cooling the brines initially at 250°C to a temperature of 4°C representative of the bottom of the seas. a,** free CO_2_ partial pressure (here 94 MPa at the end of the reaction). **b,** CO_2_ partial pressure of 10^-3.44^ MPa. **c,** CO_2_ partial pressure 1.01325 MPa. **d,** CO_2_ partial pressure of 10.1325 MPa. The amount of minerals formed is given in wt%. Talc is considered at thermodynamic equilibrium.

**Table S5: Mass of halite precipitated at depth after the serpentinization and mass of salt precipitated at the bottom of the seafloor from the hot brines once the seawater cools them.** Talc is not considered at thermodynamic equilibrium.

| pCO_2_ | free | atm | 1 atm* | 10 atm | free | atm | 1 atm | 10 atm | free | atm | 1 atm | 10 atm |
| --- | --- | --- | --- | --- | --- | --- | --- | --- | --- | --- | --- | --- |
| Temperature | 25 | 25 | 25 | 25 | 150 | 150 | 150 | 150 | 250 | 250 | 250 | 250 |
| Halite after serpentinization for 1000 kg of initial seawater (g) | 26624 | 26618 | 0 | 0 | 27425 | 27422 | 27422 | 27422 | 20739 | 21016 | 20806 | 20795 |
| Σsalts on the seafloor for 1000 kg of initial seawater (g) | 37 | 419 | 0 | 0 | 3157 | 3117 | 3117 | 2982 | 8763 | 8284 | 8675 | 8722 |

* 1 atm = 1.01325 MPa

**Table S6: Mass of halite precipitated at depth after the serpentinization and mass of salt precipitated at the bottom of the seafloor from the hot brines once the seawater cools them.** Talc is considered at thermodynamic equilibrium.

| pCO_2_ | free | atm | 1 atm* | 10 atm | free | atm | 1 atm | 10 atm | free | atm | 1 atm | 10 atm |
| --- | --- | --- | --- | --- | --- | --- | --- | --- | --- | --- | --- | --- |
| Temperature | 25 | 25 | 25 | 25 | 150 | 150 | 150 | 150 | 250 | 250 | 250 | 250 |
| Halite after serpentinization for 1000 kg of initial seawater (g) | 24546 | 23768 | 0 | 0 | 26693 | 26693 | 26692 | 26730 | 20018 | 19459 | 19886 | 20115 |
| Σsalts on the seafloor for 1000 kg of initial seawater (g) | 96 | 286 | 0 | 0 | 3104 | 3078 | 3080 | 3107 | 8557 | 9199 | 8870 | 8501 |

* 1 atm = 1.01325 MPa

1. Corresponding author. E‑mail address: [m.debure@brgm.fr](mailto:m.debure@brgm.fr) (M. Debure). [↑](#footnote-ref-1)
